# Development of patient-derived xenograft models from a spontaneously immortal low-grade meningioma cell line, KCI-MENG1

**DOI:** 10.1186/s12967-015-0596-8

**Published:** 2015-07-15

**Authors:** Sharon K Michelhaugh, Anthony R Guastella, Kaushik Varadarajan, Neil V Klinger, Prahlad Parajuli, Aamir Ahmad, Seema Sethi, Amro Aboukameel, Sam Kiousis, Ian M Zitron, Salah A Ebrahim, Lisa A Polin, Fazlul H Sarkar, Aliccia Bollig-Fischer, Sandeep Mittal

**Affiliations:** Department of Neurosurgery, Wayne State University, 4160 John R Street, Suite 930, Detroit, MI 48201 USA; Department of Pathology, Wayne State University, Detroit, MI 48201 USA; Department of Oncology, Wayne State University, Detroit, MI 48201 USA; Karmanos Cancer Institute, Detroit, MI 48201 USA

**Keywords:** G-banding karyotype, Telomerase activity, Array comparative genomic hybridization, Tumorigenicity, Progressive recurrence, Orthotopic meningioma model

## Abstract

**Background:**

There is a paucity of effective therapies for recurrent/aggressive meningiomas. Establishment of improved in vitro and in vivo meningioma models will facilitate development and testing of novel therapeutic approaches.

**Methods:**

A primary meningioma cell line was generated from a patient with an olfactory groove meningioma. The cell line was extensively characterized by performing analysis of growth kinetics, immunocytochemistry, telomerase activity, karyotype, and comparative genomic hybridization. Xenograft models using immunocompromised SCID mice were also developed.

**Results:**

Histopathology of the patient tumor was consistent with a WHO grade I typical meningioma composed of meningothelial cells, whorls, and occasional psammoma bodies. The original tumor and the early passage primary cells shared the standard immunohistochemical profile consistent with low-grade, good prognosis meningioma. Low passage KCI-MENG1 cells were composed of two cell types with spindle and round morphologies, showed linear growth curve, had very low telomerase activity, and were composed of two distinct unrelated clones on cytogenetic analysis. In contrast, high passage cells were homogeneously round, rapidly growing, had high telomerase activity, and were composed of a single clone with a near triploid karyotype containing 64–66 chromosomes with numerous aberrations. Following subcutaneous and orthotopic transplantation of low passage cells into SCID mice, firm tumors positive for vimentin and progesterone receptor (PR) formed, while subcutaneous implant of high passage cells yielded vimentin-positive, PR-negative tumors, concordant with a high-grade meningioma.

**Conclusions:**

Although derived from a benign meningioma specimen, the newly-established spontaneously immortal KCI-MENG1 meningioma cell line can be utilized to generate xenograft tumor models with either low- or high-grade features, dependent on the cell passage number (likely due to the relative abundance of the round, near-triploid cells). These human meningioma mouse xenograft models will provide biologically relevant platforms from which to investigate differences in low- vs. high-grade meningioma tumor biology and disease progression as well as to develop novel therapies to improve treatment options for poor prognosis or recurrent meningiomas.

**Electronic supplementary material:**

The online version of this article (doi:10.1186/s12967-015-0596-8) contains supplementary material, which is available to authorized users.

## Background

Meningiomas are the most common primary tumors of the central nervous system accounting for approximately 35.5% of all primary brain tumors [1]. The age-adjusted annual incidence rate is 7.22 per 100,000 individuals with a nearly 2.3-fold higher incidence in women. Over 100,000 cases were reported in United States between 2005 and 2009 [1]. Meningiomas are composed of neoplastic meningothelial cells derived from arachnoid cap cells [2]. The World Health Organization (WHO) classifies them into three main histologic subtypes: benign (grade I), atypical (grade II); and malignant (anaplastic) meningiomas (grade III) [3]. Current therapies involve surgery, fractionated radiation therapy, and stereotactic radiosurgery. There is an important group of patients with inoperable or incompletely resected low-grade meningiomas, in addition to the high-grade tumors, who develop recurrent disease following surgery and radiation therapy. Effective treatment options for these patients are exceedingly limited at present [4, 5].

Progress in the development of new treatments for meningioma is limited by a paucity of in vitro cell line models effectively limiting the availability of suitable in vivo models. Most of the well-characterized cell lines were isolated from malignant meningiomas [6–9]. Those from benign [10–12] or atypical [13] meningiomas have been genetically modified to generate stable, immortal cell lines. Of these artificially-immortalized benign meningioma cell lines, the most common method employed was viral transduction of cells to generate expression of the telomerase catalytic subunit (hTERT). The endogenous expression of hTERT is found in 30–50% of all benign and nearly 100% of high-grade meningiomas [14, 15]. Expression of hTERT in recurrent meningioma has also been observed [14]. Therefore, hTERT expression is a logical choice to manipulate the tumor cell biology to allow for continued in vitro cell growth. However, despite the careful characterizations described by the authors of those studies [10–13], it is difficult to know what aspects of the tumor cell biology may also have been altered that would confound the use of these cells as meningioma models. Moreover, two cell lines, MENII-1 [12] and Me3TSC [10], in addition to the hTERT, co-expression with human papillomavirus E6/E7 oncogenes and SV40 large T antigen, respectively, was required in order to attain immortalization, although these viral genes have not been associated with meningioma in vivo. Also of note, syngeneic mouse models of meningioma have been genetically engineered by conditional knockout of tumor suppressors such as neurofibromatosis 2 (NF2) [16, 17], however, as gene expression and regulation are considerably divergent between mouse and human [18], human tumor models may be more suitable for translational research purposes [19].

Meningiomas were among the first CNS solid tumors found to have consistent cytogenetic aberrations [20–22], The most well explored observation is the loss of heterozygosity due to a loss of one copy of the long arm of chromosome 22 [23, 24], and this is usually the only chromosomal loss associated with benign meningioma [25]. Atypical and malignant meningiomas also have losses of the short arm of 1 and the long arm of 14q [26], and gains of the long arms of chromosomes 1, 9, 12, 15, 17, and 20 [25, 27]. These striking chromosomal abnormalities may be related to the hTERT expression and telomerase activity [28] found in some benign and almost all high-grade meningiomas [14, 15].

While there have been numerous studies examining the genetic alterations characteristic of meningiomas, these have yielded little in the way of efficacious treatment alternatives. As such, there is a critical need for development of pre-clinical tumor models to improve the understanding of the underlying pathobiology of meningiomas and for the development and testing of novel therapeutic approaches. Human cell culture systems represent an essential experimental tool. However, most studies use primary, early passage human meningioma cell lines that typically senesce after a few passages. Here, we report the isolation and characterization of a novel, spontaneously-immortalized cell line, which we have designated as KCI-MENG1, derived from a WHO grade I benign meningioma and used to develop mouse xenograft models.

## Methods

### Original tumor specimen

A 46-year-old woman with an olfactory groove WHO grade I meningioma underwent surgical resection. Tumor samples were obtained immediately following surgical resection after adequate material was reserved for histopathological diagnosis. The specimen was divided into multiple pieces. One piece was frozen on dry ice and subsequently stored at −80°C, and another was dissociated for in vitro cultures. The study was approved by the Wayne State University Institutional Review Board and written informed consent was obtained from the patient.

### Isolation and culture of primary human meningioma cells

The tumor sample was washed in phosphate-buffered saline (PBS) with 2 mM ethylenediaminetetraacetic acid (EDTA) to remove blood and then chopped into fragments (<1 mm) using a sterile single-edge razor blade. The fragments were washed in PBS without EDTA and digested with collagenase type IV (0.5 mg/ml in PBS; Sigma-Aldrich, St. Louis, MO, USA) for 30–60 min at 37°C with occasional mixing. A single cell suspension was prepared by trituration with a 5 ml pipet. KCI-MENG1 cells were cultured in DMEM/F12 supplemented with 2× non-essential amino acids, 10 µg/ml gentamicin (Sigma-Aldrich) and fetal bovine serum (10% v/v; Life Technologies, Carlsbad, CA, USA), in a humidified atmosphere of 5% CO_2_/air. Culture media was changed 2–3 times per week. Cell growth was monitored by inspection with an inverted microscope.

### Growth kinetics

The doubling times of both low passage (P6 and P9) and high passage (P72) KCI-MENG1 cells were determined by counting cells at multiple time points after culture seeding. On Day 0, 1,000 viable cells/well were seeded in 96-well plates and fed with the above culture medium. Cultured cells were harvested with Accumax (Innovative Cell Technologies, San Diego, CA, USA) and counted with a Beckman Coulter counter (Beckman Coulter, Inc., Indianapolis, IN, USA) at several time points (ranging from 18 to 96 h) after plating (n = 3 wells at each time point). The growth curves were plotted and doubling times calculated with GraphPad Prism v6.04 (GraphPad Software, Inc., La Jolla, CA, USA) using the exponential growth equation and one-way ANOVA with Tukey’s multiple comparisons test.

### Immunohistochemistry of primary tumor and xenograft mouse tumor

The original tumor was processed for the usual markers used for clinical diagnosis of meningioma. Tissue sections (5 µm) were cut from the selected formalin-fixed paraffin-embedded tumor block and mounted on charged slides and used for immunohistochemistry (IHC) analysis using specific antibodies for epithelial membrane antigen (EMA), progesterone receptor (PR), Ki-67, E-cadherin, N-cadherin, and vimentin. Standard IHC protocols using avidin–biotin complex were used as previously described [29, 30]. A standard protocol for diagnostic hematoxylin and eosin (H&E) staining was also performed. The IHC protocol was optimized for antigen retrieval and antibody dilution and incubation conditions. Briefly, after deparaffinizing and hydrating with PBS (pH 7.4), the sections were pretreated with hydrogen peroxide (3%) for 10 min to remove endogenous peroxidase activity, followed by antigen retrieval via steam bath for 20 min in EDTA. Primary antibody was applied, followed by washing and incubation with the biotinylated secondary antibody for 30 min at room temperature. After another set of washes, avidin-peroxidase was added allowing for detection of antibody binding using the substrate diaminobenzidine. Sections were counterstained with Mayer hematoxylin, dehydrated, and mounted for microscopic examination.

The xenograft mouse tumor tissue sections underwent similar staining protocols except antigen retrieval was not performed, and sections stained with mouse-derived primary antibodies were stained with the Mouse-on-Mouse™ Immunodetection Kit (Vector Labs, Burlingame, CA, USA) using the manufacturer’s protocol except that the secondary antibody solution was prepared with only 1 µl of secondary antibody instead of 10 µl.

### Immunocytochemistry of primary tumor cells and xenograft mouse tumor cells

KCI-MENG1 cells or KCI-MENG1-LPSX cells (dissociated cells from second generation xenograft mouse tumor) were seeded onto Millicell^®^ EZ slides (EMD Millipore, Billerica, MA, USA) and fixed with 4% paraformaldehyde before proceeding with immunostaining procedures using either the mouse or rabbit VECTASTAIN^®^ Elite ABC kit (Vector Labs) following the manufacturer’s protocol. Primary antibodies used targeted the following proteins: EMA (cat.#247M-94), PR (cat.#323R-14), Ki-67 (cat.#275R-14), vimentin (cat.#347R-14; all from CellMarque, Rocklin, CA, USA), and N-cadherin (cat.#NBP1-48309, Novus Biologicals, Littleton, CO, USA). All primary antibodies were used at a 1:100 dilution. The peroxidase substrate used was Vector ImmPACT^®^ DAB solution (cat.#SK-4105, Vector Labs) and sections were mounted with VectaMount™ (cat.#H-500, Vector Labs).

### Telomerase activity

Telomerase activity was measured using the TRAPeze^®^ RT Telomerase Detection Kit (EMD Millipore, Billerica, MA, USA) as described by the manufacturer. Protein concentrations of lysed cells samples were measured by the bicinchoninic acid protein assay using bovine serum albumin as a standard (Thermo Scientific, Wilmington, DE, USA). Real-time PCR was performed with a StepOnePlus™ Real-Time PCR System (Life Technologies, Grand Island, NY, USA). A standard curve was generated with DNA standards of known abundance. Controls for the DNA polymerase activity and a positive control cell line known to have high telomerase activity were also included. Only samples falling within the linear range of detection were included in the data analysis, and all samples were normalized to the amount of protein included in the reaction. All samples were assayed in triplicate. ANOVA with Tukey’s multiple comparisons test was performed.

### Cytogenetic analysis

Cultured cells were used to prepare chromosomes for karyotyping per a previously described method [31]. Briefly, using aseptic techniques, cells were incubated with 10 µg/ml colcemid in media at 37°C for 45 min. Cells were harvested and centrifuged. The supernatant was removed and resuspended cells were treated with pre-warmed 0.075 M KCI, added slowly with agitation, and incubated at 37°C water bath for 12–20 min. Fixative was added to the tube which was mixed by gentle inversion and centrifuged. The supernatant was removed and the pellet resuspended and fresh fixative was slowly added to a total volume of 10 ml. The cell suspension was mixed and incubated at −20°C for 1 h. At the end of the incubation period, 2–3 drops of cell suspension were placed on a microscope slide and allowed to dry at room temperature. The quality of the cell preparation was checked under phase contrast microscopy before slides were analyzed for G-banding with Giemsa dye. At least 20 metaphases were analyzed for each cell passage sample. All chromosomal abnormalities are reported in accordance with the current international standard nomenclature [32].

### Genomic analysis

DNA was extracted from a fresh-frozen piece of the original tumor (23 mg wet weight) and from KCI-MENG1-LP (P6) and KCI-MENG1-HP (P86) cells using resin-based purification techniques. DNA samples were quantified by NanoDrop (Thermo Scientific). Array comparative genomic hybridizations (aCGH) were performed with Agilent SurePrint G3 ISCA CGH+SNP 180K microarrays (Agilent Technologies, Santa Clara, CA, USA) using a commercially-available, genetically-normal female DNA standard. DNA samples were labeled with the SureTag Labeling Kit (Agilent). Bioinformatics analysis was performed using Agilent CytoGenomics Edition 2.5.8.1 with the significance threshold set at 10; log_2_ ratio cutoffs ≥0.3 and ≤0.37 according to the laboratory validated reproducibility measures. Data were further processed by filtering against the Cancer Gene Census (Wellcome Trust Sanger Institute, Genome Research Limited, Hinxton, UK) [33] to identify genes with well-characterized roles in cancer.

### Generation of mouse xenograft meningioma tumors

All animal experimental protocols were approved by the Wayne State University Institutional Animal Care and Use Committee. Low passage KCI-MENG1-LP cells were cultured as described above. When cultures reached confluence, P9 cells were harvested for injection into ICR SCID mice (spontaneous mutant T- and B cell deficient mice; Taconic, Hudson, NY, USA). Cells were washed and resuspended in PBS and injected subcutaneously into the mouse flank bilaterally (2 × 10^7^ cells/injection). After ~4 weeks, the xenograft tumor reached an estimated mass of 1 g. Animals were sacrificed and the harvested tumor tissue was cut into ~30–40 mg fragments and implanted bilaterally into naïve SCID mice. After ~6 weeks, these second generation tumors (KCI-MENG1-LPSX) had each grown to an estimated mass of 1.4–1.6 g. After sacrifice, a third generation of mice (M3; SCID/NCr (BALB/C background) from the NIH-Frederick Cancer Research, Frederick, MD, USA) was implanted with tumor fragments, and the remaining tumor tissue was divided into pieces. The tumor pieces were: (1) flash-frozen and stored at −80°C; (2) fixed with 4% paraformaldehyde or 10% formalin; and (3) dissociated into a single cell suspension using the gentleMACS Dissociator™ and Human Tumor Dissociation Kit (Miltenyi Biotech, San Diego, CA, USA) following the manufacturer’s protocols. These dissociated cells, termed KCI-MENG1-LPSX-CL, were cultured and analyzed as described above. In addition, we also performed subcutaneous injections with high passage (P72) KCI-MENG1-HP cells into the SCID/NCr mice (3 × 10^6^ cells/injection with BD Matrigel™ Basement Membrane Matrix, BD Biosciences, San Jose, CA, USA) and completed the same procedures as above with the resulting tumor tissues and the resulting cell line termed KCI-MENG1-HPSX-CL.

For the orthotopic mouse model, stereotactic brain injections were performed with the Just For Mice™ Stereotaxic Instrument and the Nanomite Programmable Syringe Pump (Harvard Apparatus, Holliston, MA, USA). The cranium was exposed and a burr hole was drilled 1 mm anterior of bregma and 1 mm lateral from midline using a #3 ball mill tip with the Micro-Drill System (Harvard Apparatus, Holliston, MA, USA). KCI-MENG1-LPSX-CL cells were suspended in RPMI media (1 × 10^6^ cells/10 µl). Either 5 or 10 µl of the cell suspension were injected 0.5 mm subdural. Post-operatively, mice were monitored for overall health 2–3 times per week. Magnetic resonance imaging (MRI) with gadolinium contrast was performed 4 weeks post-injection. Mice were euthanized and brain and tumor tissues collected. IHC was performed as described above.

**Figure 1:**
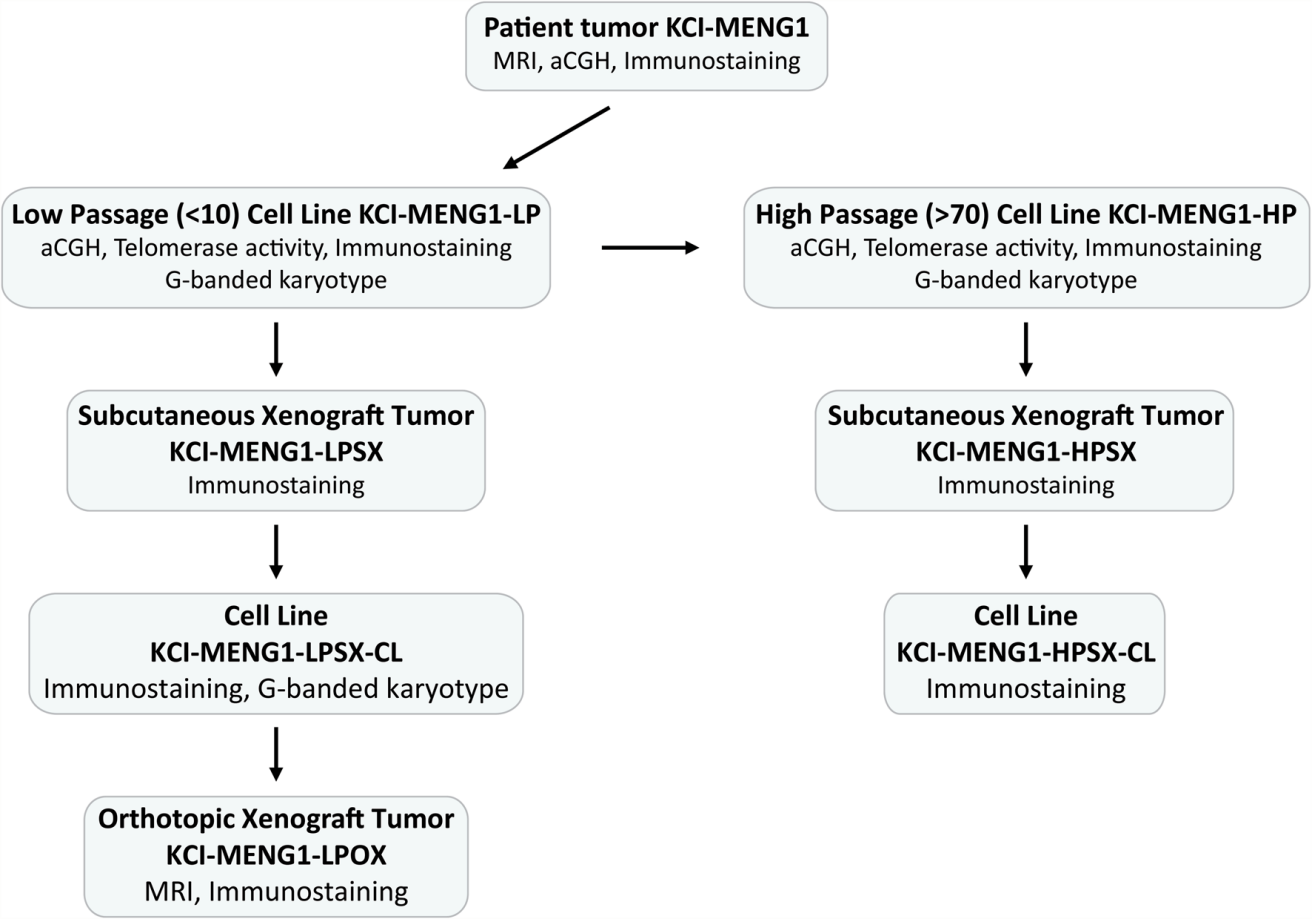
Flowchart describing the generation of in vitro and in vivo models from the KCI-MENG1 patient tumor.

Figure 1 outlines the workflow for the generation of various cell lines and xenograft mouse models.

## Results

### Neuroimaging and histopathological findings of original tumor

**Figure 2:**
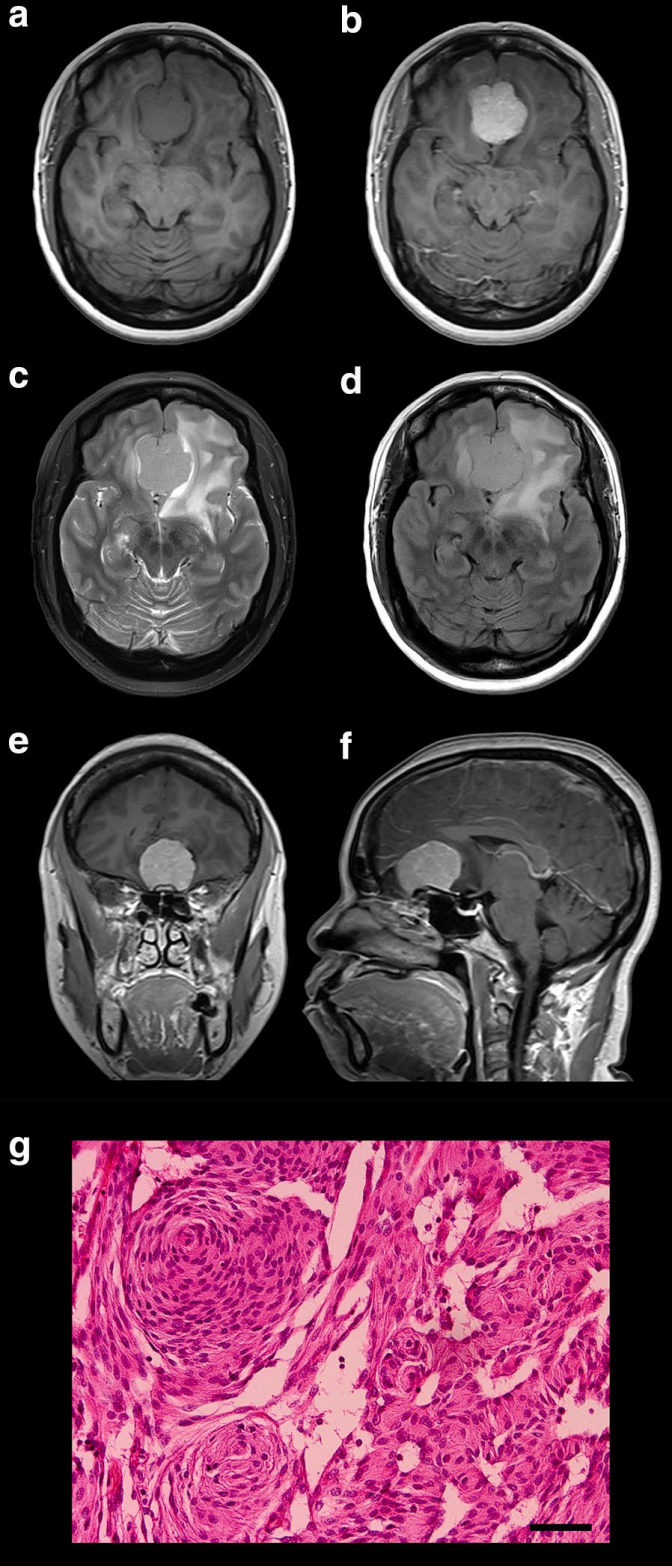
Neuroimaging and histopathological findings of original KCI-MENG1 tumor. MRI showed a well-circumscribed (**a**) homogeneously-enhancing (**b**, **e**, **f**) 3.7 × 3.7 × 2.6 cm olfactory groove meningioma with significant peritumoral vasogenic edema (**c**, **d**). H&E staining revealed neoplastic proliferation of moderately cellular meningothelial cells with several whorls and occasional psammoma bodies (**g**) consistent with a WHO grade I benign meningioma. *Scale bar* 50 µm.

**Figure 3:**
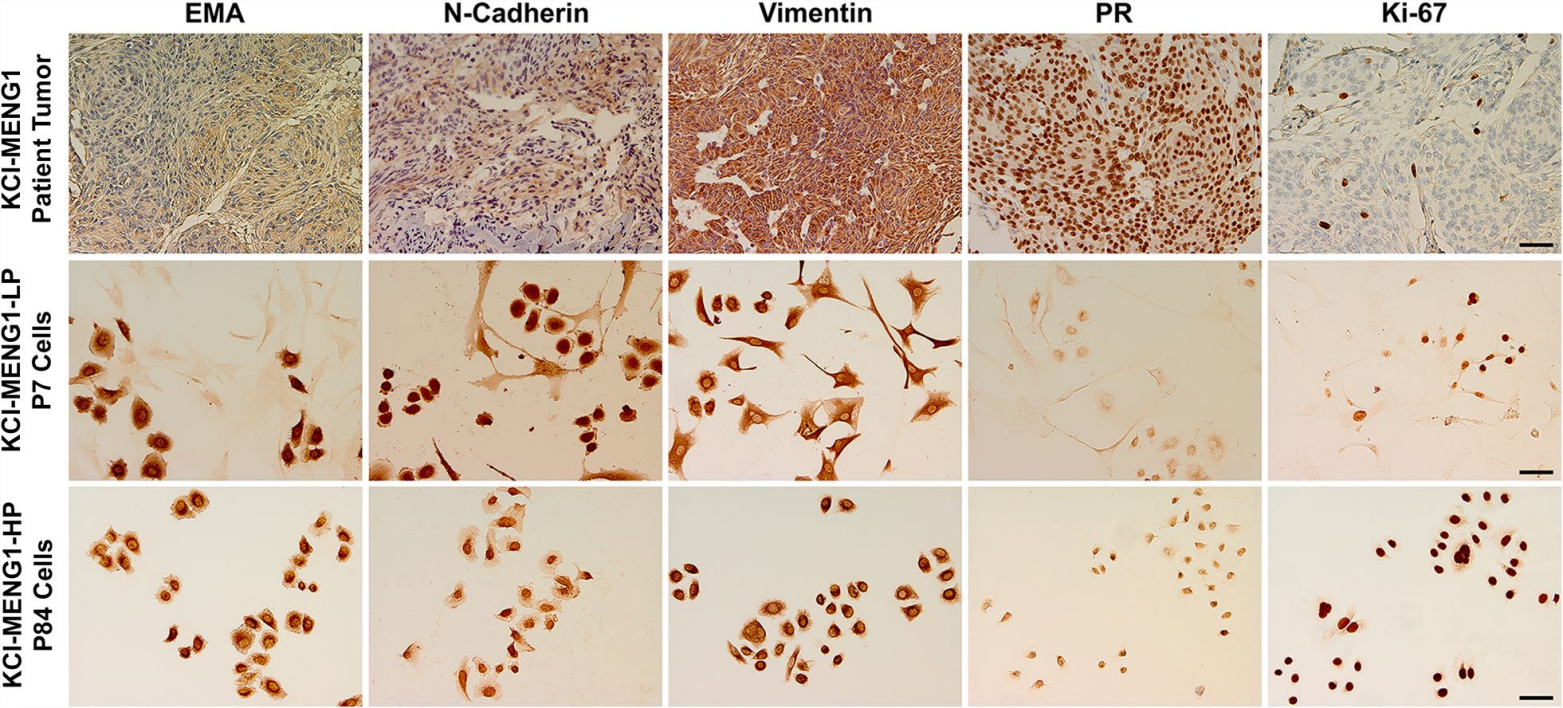
Immunostaining of original tumor, low passage, and high passage KCI-MENG1 cells. The original patient-derived tumor (*top row*) showed moderate and patchy immunoreactivity for epithelial membrane antigen (EMA); strong and diffuse immunostaining for progesterone receptor (PR); and a Ki-67 proliferative index of 2–3%. There was also strong immunostaining for N-cadherin and vimentin. KCI-MENG1-LP cells (*middle row*) and KCI-MENG1-HP cells (*bottom row*) maintained expression of EMA, N-cadherin, and vimentin but had significantly reduced PR expression compared to the original tumor. Whereas Ki-67 labeling was found in only a small number of cells in the original tumor and low passage cells, it was positive in virtually all P84 cells. *Scale bar* 50 µm.

**Figure 4:**
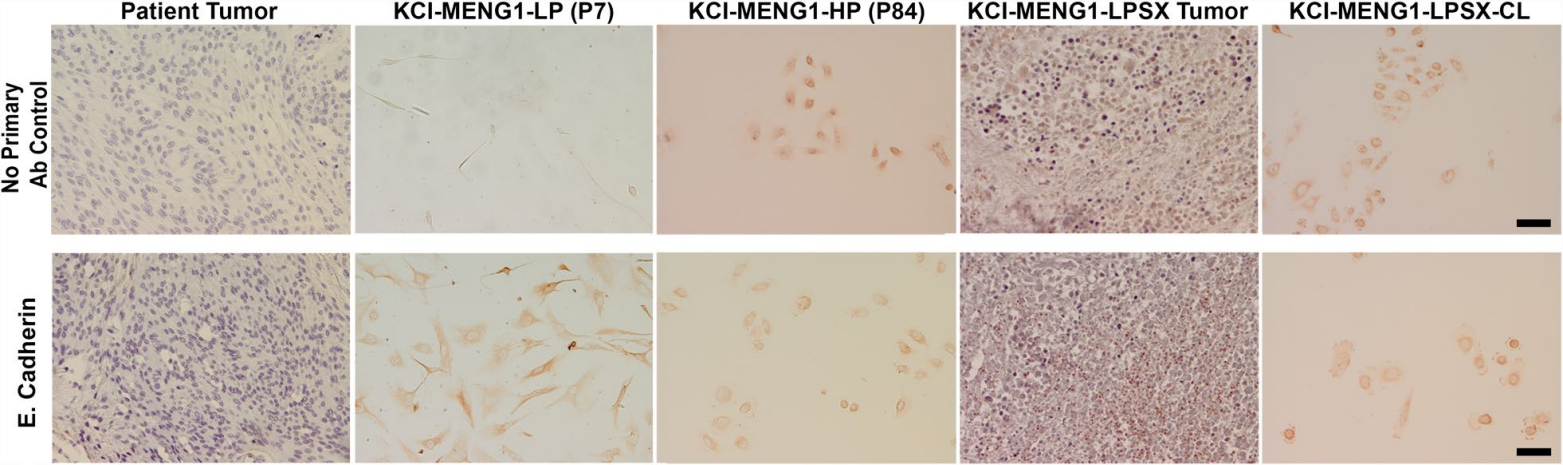
Immunostaining of original patient tumor, low and high passage KCI-MENG1 cells, and subcutaneous xenograft tumor. The original patient-derived tumor showed moderate immunoreactivity for E-cadherin which was maintained in all in vitro and in vivo models. *Scale bar* 50 µm.

High-resolution 3T MRI of the patient’s brain revealed a well-circumscribed avidly-enhancing extraaxial anterior cranial fossa mass consistent with an olfactory groove meningioma (Figure 2a–f). The mass measured 3.7 × 3.7 × 2.6 cm in size and was associated with significant peritumoral vasogenic edema. The patient underwent a gross total resection of the tumor (Simpson grade I). Histopathological analysis of the firm tumor was consistent with a WHO grade I typical meningioma composed of moderately cellular meningothelial cells with several whorls and occasional psammoma bodies (Figure 2g). The tumor cells showed moderate and patchy immunoreactivity for EMA; strong and diffuse immunostaining for PR; and a Ki-67 proliferative index of 2–3%. Furthermore, mesenchymal markers were also detected. Strong staining for the cytoskeletal protein vimentin and moderate staining for the cell adhesion molecule N-cadherin were observed (Figure 3, top row), with absent staining for E-cadherin (Figure 4).

### KCI-MENG1 morphologic, growth, and immunocytochemical characteristics

**Figure 5:**
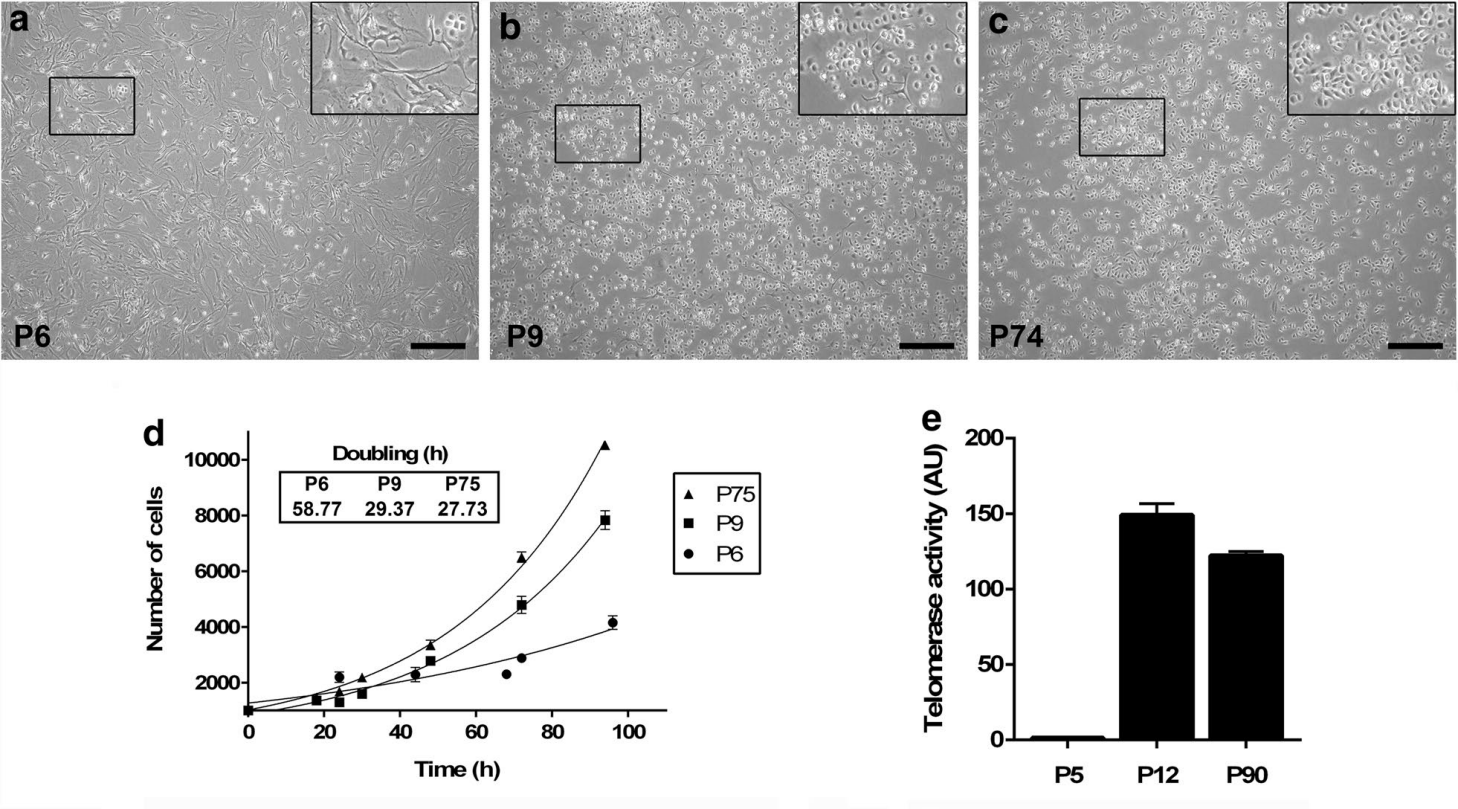
Morphology, growth characteristics, and telomerase activity of primary cell cultures. In P6 KCI-MENG1-LP cells, the spindle-shaped cells account for the majority the cell population (**a**). In contrast, the round cells become more predominant at P9 with much fewer spindle cells (**b**). At higher passages (**c**), KCI-MENG1-HP cultures are composed of exclusively round-shaped cells. This was also reflected in the growth curves of the low- vs. high passage cells (**d**). The P6 cells have a linear and shallow growth curve that was maintained for 96 h after cultures were seeded. P9 and P75 cells both demonstrated biphasic growth curves, with the shift in slope becoming apparent after 72 h (ANOVA *p* < 0.001). Likewise, the telomerase activity in P5 cells was very low, whereas it was very high in both P12 and P90 cells (ANOVA *p* < 0.0001) (**e**). *Scale bar* 50 µm.

KCI-MENG1-LP cells have two prominent cell morphologies, spindle and round, whereas the KCI-MENG1-HP are homogeneously round (Figure 3, middle and bottom rows, Figure 5a–c; summarized in Table 1). At P6, the majority of cells are spindle-shaped, while at P9, the round cells are predominant with relatively few spindle cells. This alteration in the relative abundance of the two cell morphologies as the cells were passaged was also reflected in the cell growth rates. The P6 cells have a linear and shallow growth curve that was maintained for 96 h after cultures were seeded. P9 and P75 cells both demonstrated biphasic growth curves, with the shift in slope becoming apparent after 72 h (Figure 5d).

**Table 1:** Meningioma cell lines reported in the literature

Cell line	References	Source	Manipulation	IHC	Morphology	Telomerase	Cytogenetics	Genomics	Xenograft tumor
KT21-MG1	Tanaka et al. [8]	Grade III	None	Vimtentin+ GFAP−	Round and spindle	n/a	Shows loss of chromosome 22	Southern blotting	Grew
IOMM-Lee	Lee [6]	Grade III	None	Vimentin+ S-100− Estrogen receptor− GFAP− FVIII-RA− EMA− NSE− Leukocyte common antigen− Keratin− Actin− Neurofilaments−	Mostly round, some spindles though	n/a	45–65 chromosomes, model number of 49, no loss of chromosome 22	n/a	Grew, but no indication as to the method of injection
MENII-1	Striedinger et al. [13]	Grade II	Telomerase expression and HPV E6/E7	Merlin+ YAP	Round	n/a	n/a	n/a	n/a
CH-157MN	Tsai et al. [34]	Unknown grade	n/a	VEGF+	Spindles	n/a	n/a	n/a	n/a
HKBMM	Ishiwata et al. [9]	Grade III	n/a	Desmin+ PKK-1+ S-100− EMA− Vimentin+	Spindles	n/a	Model number of 48, 21p+, aneuploidy	n/a	Subcutaneous
BEN-MEN-1	Puttmann et al. [11]	Grade I	hTERT	GFAP− Vimentin+ EMA+ PR− Estrogen Rec− Cytokeratins− Ki-67−	Mostly spindles, few scattered round cells	RT-PCR, TRAP assay and Southern blotting	Loss of one chromosome 22 in all cells, while other chromosomal changes were absent (45, XX, 22)	n/a	Subdural (subarachnoidal)
F5	Yazaki et al. [7]	Grade III	n/a	S-100+ Vimentin+	n/a	n/a	Loss of chromosome 22	n/a	Subcutaneous and intracranial
Me10T	Cargioli et al. [10]	Grade I	hTERT	EMA− PR− Vimetin+ S-100+ Cytokeratin+	Round and spindle	Discussed, no data—transduced cells showed activity whereas the non-transduced cells did not	Monosomy only of chromosome 22	n/a	Intracranial—subdural
Me3TSC	Cargioli et al. [10]	Grade I	hTERT and SV40 large T antigen	EMA− PR− Vimetin+ S-100+ Cytokeratin+	Round and spindle	Discussed, no data—transduced cells showed activity whereas the non-transduced cells did not	Monosomy for chromosome 22, deletions in chromosomes 9 and 11, and translocations between 1 and 5	n/a	Intracranial—subdural
SF3061-Parental	Baia et al. [12]	Grade III	hTERT	Vimentin+ Desmoplakin+	Spindle	Quantitative PCR	Subset of the losses in the primary tumor: 9p24-p21; 11q23-qtel; 13q12- q21; 17p	aCGH	n/a
SF4433-Parental	Baia et al. [12]	Grade I	E6/E7-hTERT	Vimentin+ Desmoplakin+	Round and spindle	Quantitative PCR	No chromosomal abnormalities found	aCGH	n/a
SF4068- Parental	Baia et al. [12]	Grade I	E6/E7-hTERT	Vimentin+ Desmoplakin 1 and 2+ NF2−	Spindle	Quantitative PCR	Gain of chromosome 5p and loss of chromosome 15	aCGH	n/a
KCI-MENG1	Michelhaugh (2015-this study)	Grade I	None	EMA+ Vimentin+ N. Cadherin+ PR− Ki-67+ E. Cadherin−	Low passage: heterogeneous for round and spindle cells High passage: homogeneous for round cells	TRAP assay	Near triploid, multiple translocations	aCGH	Subcutaneous Intracranial—subdural

To further characterize the KCI-MENG1-LP vs. KCI-MENG1-HP cells, the telomerase activity was measured with a highly sensitive real-time PCR assay. As shown in Figure 5e, P5 cells had very little telomerase activity, whereas the telomerase activity in both P12 and P90 cells was highly robust. Immunostaining of the low- and high passage cells (Figure 3, middle and bottom rows) revealed that the in vitro cultured cells maintained expression of EMA, N-cadherin, and vimentin, and also were negative for E-cadherin (Figure 4) as was the original tumor (Figure 3, top row). Closer examination of the EMA panel for the low passage cells suggests that the positive EMA staining is found predominantly in the round cells and only weakly in the spindle cells, which is congruent with the moderate immunostaining observed in the original tumor. PR expression in the cultured cells is dramatically reduced compared to the original tumor. The Ki-67 labeling, which is indicative of the cells’ proliferative activity, is found in a relatively small number of cells in the original tumor and in the low passage culture, however, the Ki-67 staining in the high passage cells is very intense in virtually all the cells assayed.

### Cytogenetic analysis

**Figure 6:**
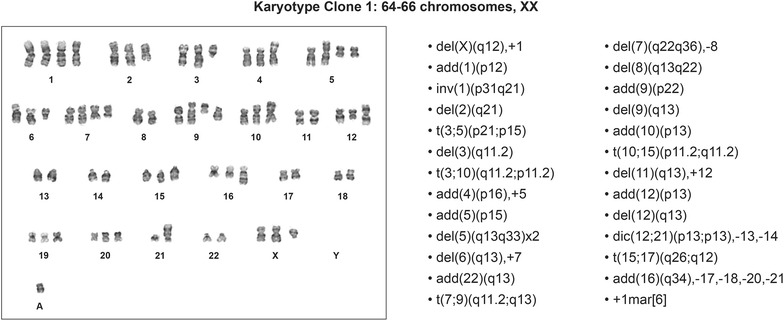
G-banded karyotype from KCI-MENG1-LP cell line showing numerous structural and numerical chromosomal aberrations. This near triploid karyotype was identified in 6 out of 20 metaphases examined at KCI-MENG1-LP cells. The identical karyotype was identified in 20 out of 20 metaphases examined in KCI-MENG1-HP cells.

G-banded karyotyping can detect microscopic genomic abnormalities such as chromosomal inversions, duplications, deletions, balanced and unbalanced translocations, as well as more general aneuploidies [35]. In our study, G-banded karyotyping of 20 metaphases from KCI-MENG1-LP at P4 revealed an abnormal female karyotype in ten metaphases. The other ten metaphases were normal. The abnormal metaphases had two distinct unrelated clones. Clone 1, found in six metaphases, had a near triploid karyotype containing 64–66 chromosomes with numerous structural and numerical chromosomal aberrations as listed in the karyotype (Figure 6). In clone 2, four metaphases had t(2;13)(q37;q22) and t(4;7)(q21;p13) [4] translocations. Chromosome analysis of KCI-MENG1-HP (P86) revealed that all 20 metaphases examined had the clone 1 near triploid karyotype containing 64–66 chromosomes with numerous structural and numerical chromosomal aberrations as observed in P4 cells. Similarly, KCI-MENG1-LPSX-CL cells derived from the second generation mouse xenograft tumor also demonstrated this near-triploid clone 1 karyotype in 20 out of 20 metaphases examined. Neither of these clones demonstrated a loss of part or all of chromosome 22.

### Genomic analysis

To assess the submicroscopic genomic abnormalities, aCGH was performed on a fresh frozen piece of the original KCI-MENG1 tumor specimen, KCI-MENG1-LP, and KCI-MENG1-HP cells. Data generated from the aCGH was filtered using the Sanger Cancer Gene Census (listed in Additional file 1: Table S1) to focus our attention on only those genes with a clearly established role in any cancer type. Using this filter, we found no amplified or lost genes from the original tumor specimen. Therefore, this tumor had no loss of the NF2 tumor suppressor gene. For both the low- and high passage cells, many gene amplifications were identified, though very few deletions. Genes found to be amplified at the level of 0.5 or higher in the KCI-MENG1-LP cells are shown for both passages in Table 2, with the complete aCGH dataset available in Additional file 2: Table S2. Comparing the two cell passages, there is approximately a doubling of all the amplifications in the KCI-MENG1-HP cells. Likewise, the gene deletion shown at the bottom of Table 2 shows a more robust loss in the KCI-MENG1-HP cells. Moreover, in the high passage cells, many of the gene amplifications are congruent with long arm gains of chromosomes 1, 9, 12, 15, 17, and 20.

**Table 2:** Array comparative genomic hybridization (aCGH) data in low- and high-passage KCI-MENG1 cells

Chromosome	CytoBand	GeneID	Gene name	Amplification P6	Deletion P6	Amplification P86	Deletion P86
chr5	p15.33 - p11	IL7R	Interleukin 7 receptor	1.213931		2.050158	
chr5	p15.33 - p11	LIFR	Leukemia inhibitory factor receptor alpha	1.213931		2.050158	
chr11	q21 - q22.2	BIRC3	Baculoviral IAP repeat containing 3	0.899188		1.654617	
chr11	q14.3 - q22.2	BIRC3	Baculoviral IAP repeat containing 3	0.709864		1.472601	
chr11	q14.3 - q22.2	MAML2	Mastermind-like 2 (Drosophila)	0.709864		1.472601	
chr11	q14.1 - q14.2	PICALM	Phosphatidylinositol binding clathrin assembly protein	0.709864		1.233367	
chr3	q26.1 - q26.2	MECOM	MDS1 and EVI1 complex locus	0.702415		1.47986	
chr11	p11.2 - p11.12	DDB2	Damage-specific DNA binding protein 2, 48 kDa	0.675591		1.367689	
chr10	q11.21 - q22.2	KAT6B	K(lysine) acetyltransferase 6B	0.499694		1.213813	
chr10	q11.21 - q22.2	NCOA4	Nuclear receptor coactivator 4	0.499694		1.213813	
chr10	q11.21 - q22.2	PRF1	Perforin 1 (pore forming protein)	0.499694		1.213813	
chr10	q11.21 - q22.2	TET1	Tet methylcytosine dioxygenase 1	0.499694		1.213813	
chr19	p13.3	STK11	Serine/threonine kinase 11		−1.376387		−3.097343

### Tumorigenicity in SCID mice: morphological, immunohistochemical, and cytogenetic analysis

**Figure 7:**
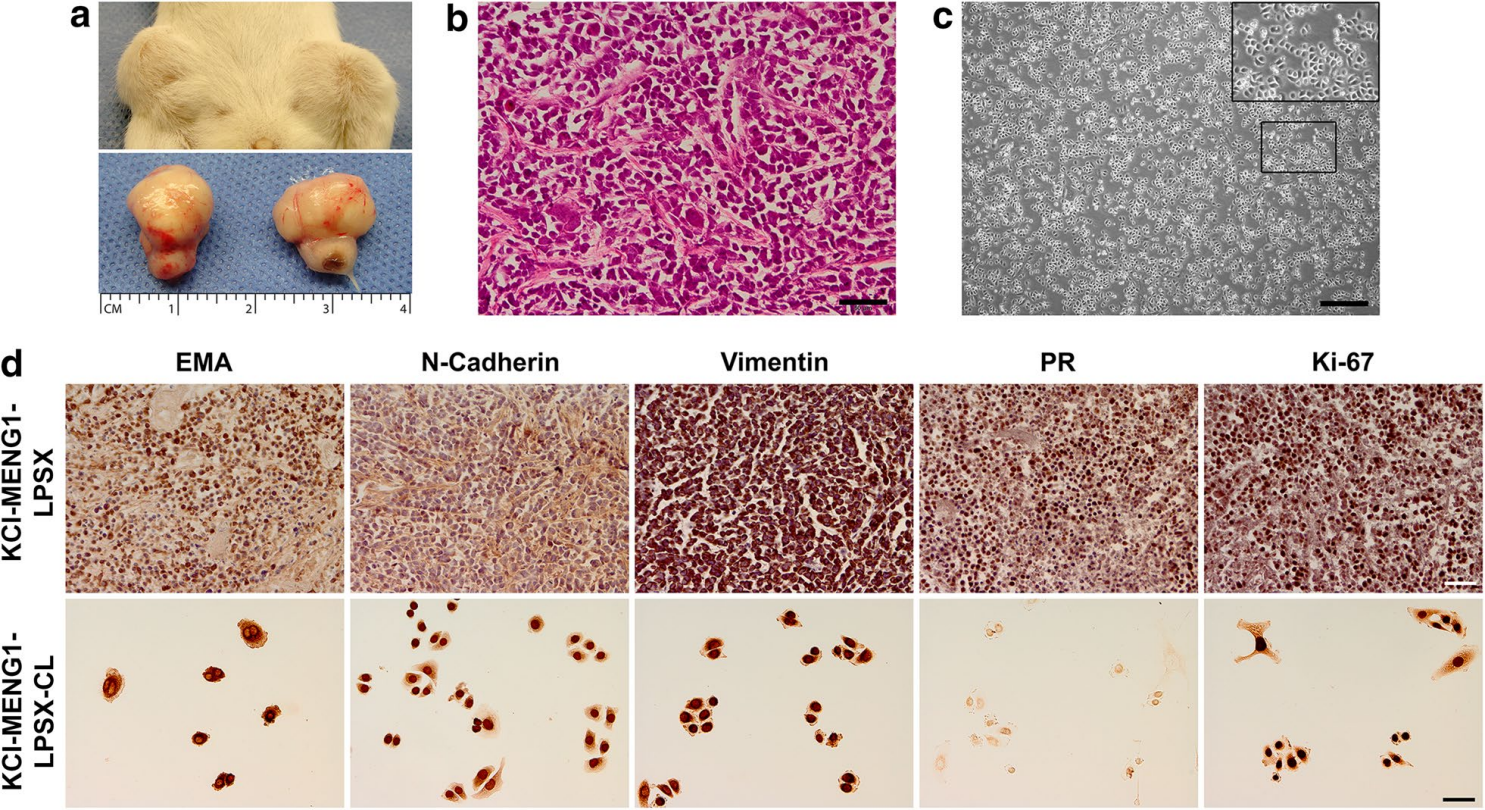
Human meningioma mouse xenograft model KCI-MENG1-LPSX generated with the spontaneously immortal cell line KCI-MENG1-LP. Tumors from immunocompromised SCID mice were dissected (**a**) and the derivative cell line KCI-MENG1-LPSX CL was generated. The H&E staining of the mouse tumor revealed a pattern of moderately cellular meningothelial cells similar to the original patient tumor (**b**). The KCI-MENG1-LPSX CL cells were composed of the round-shaped cells similar to the high passage parent cell line KCI-MENG1-HP (**c**). The EMA, PR, and N-cadherin IHC of the mouse tumor highly resembled the original patient-derived tumor (**d** *top row*). The vimentin- and Ki-67-stained cells in the mouse tumor tissue were markedly more abundant and more intensely stained than in the original tumor (**d** *top row*). KCI-MENG1-LPSX CL cells displayed the same patterns of immunostaining as the high passage parent cell line KCI-MENG1-HP, including the loss of PR staining (**d** *bottom row*). *Scale bar* 50 µm.

**Figure 8:**
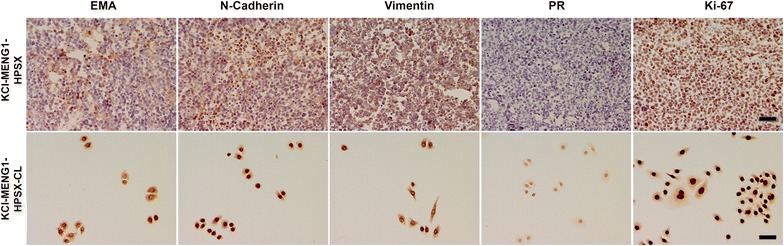
KCI-MENG1-HPSX high passage mouse tumor and cell line (KCI-MENG1-HPSX CL). IHC revealed a similar staining pattern as compared to the KCI-MENG1-LPSX tumor and KCI-MENG1-LPSX cell line, with the exception of loss of PR in the HPSX tumor. *Scale bar* 50 µm.

ICR SCID mice, which are both T- and B-cell deficient, were used for this experiment. One of the mice implanted with the second generation KCI-MENG1-LPSX is shown in Figure 7a. After sacrifice, tumors were dissected and the tissue was processed and the derivative cell line KCI-MENG1-LPSX-CL was generated (Figure 7c). In addition to H&E staining (Figure 7b), immunostaining for the usual meningioma diagnostic markers, as well as the mesenchymal markers, was performed on both the mouse tumor tissue and KCI-MENG1-LPSX-CL cells (Figure 7d). The H&E staining of the mouse tumor tissue revealed a pattern of moderately cellular meningothelial cells similar to the original tumor (Figure 2g). The EMA, PR, and N-cadherin IHC of the KCI-MENG1-LPSX tumor strongly resembled the original patient-derived KCI-MENG1 tumor. The vimentin- and Ki-67-stained cells in the mouse KCI-MENG1-LPSX tissue were markedly more abundant and more intensely stained than in the original KCI-MENG1 tumor. KCI-MENG1-LPSX-CL cells displayed the same patterns of immunostaining as the KCI-MENG1-HP cells, including the loss of PR staining. Likewise, the KCI-MENG1-LPSX-CL cells had the same aberrant karyotype and at the same frequency as the KCI-MENG1-HP cells (shown in Figure 6). Similarly, additional mice were implanted subcutaneously with 3 × 10^6^ KCI-MENG1-HP cells. These mice reached an estimated tumor burden of 1.6 g and required sacrifice 26 days post-implantation. In Figure 8, immunostaining of tumor tissue KCI-MENG1-HPSX and cells isolated from these tumors (KCI-MENG1-HPSX-CL) appeared equivalent to the staining of the low passage tumor tissue and cells shown in Figure 7 with the exception of an apparent loss of PR in the high passage KCI-MENG1-HPSX tumor.

**Figure 9:**
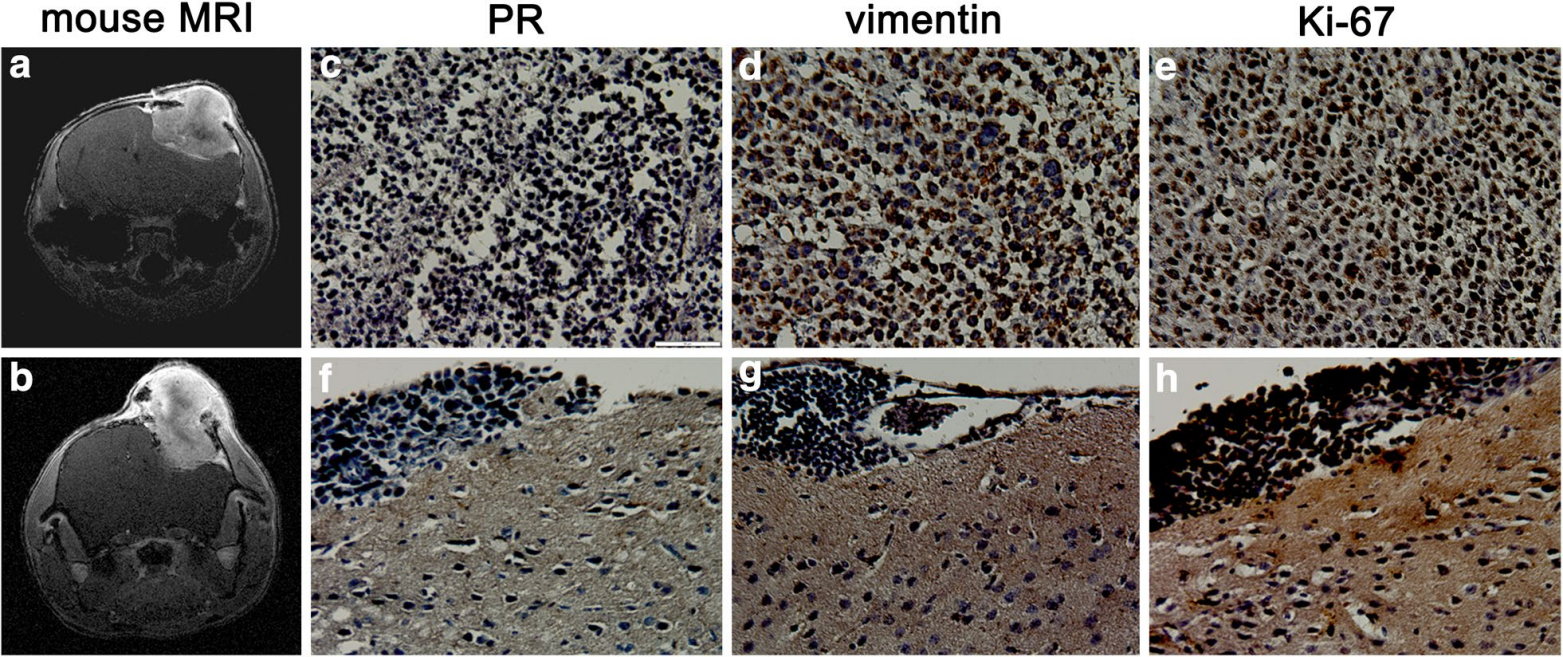
Orthotopic mouse model of human meningioma generated by subdural implantation of KCI-MENG1-LPSX CL cells. Subdural implantation of cells was performed and tumors were observed with gadolinium-contrast on MRI (**a** 0.5 × 10^6^ cells implanted; **b** 1.0 × 10^6^ cells implanted). Harvested KCI-MENG1-LPOX tumor tissue strongly stained for PR (**c**), vimentin (**d**), and Ki-67 (**e**). Tumor cells expressing PR (**f**), vimentin (**g**), and Ki-67 (**h**) are found intermingled in the adjacent brain tissue. *Scale bar* 50 µm.

Similarly, subdural implantation of KCI-MENG1-LPSX-CL cells generated gadolinium-enhancing tumors (KCI-MENG1-LPOX), with a likely necrotic core. These orthotopic tumors were strongly positive for PR, vimentin, and Ki-67. In the adjacent brain, cells with this phenotype are found intermingled within the brain parenchyma (see Figure 9).

## Discussion

Improved survival and reduced recurrence are expected following complete excision of the intracranial meningiomas [36, 37]. However, up to 5% of benign meningiomas [38] and 17–40% of atypical meningiomas recur at 5 years following complete resection [38, 39]. Not surprisingly, partial resection is associated with a significantly higher risk of tumor recurrence (87% for atypical meningioma) [37, 39]. Generally, 5-year survival rate is 95 and 61% after total and partial removal of the tumor, respectively [36, 37, 39]. Furthermore, up to 29% of recurrent benign meningiomas [26, 40] were reported to progress into more aggressive higher grades. The currently available treatment options following partial resection or recurrence of the tumor are surgery and radiotherapy [41–43]. To date, there are limited effective chemotherapeutic options for the treatment of refractory or recurrent benign or high-grade meningiomas [4, 5].

One obstacle in the development of novel therapeutic agents for meningioma treatments is a relative lack of suitable in vitro and in vivo model systems. Most cell lines originate from malignant meningiomas [6–9] or from benign [10–12] or atypical [13] meningiomas that have been genetically modified for immortalization (see Table 1). In this manuscript, we have described KCI-MENG1, a native, apparently immortal cell line derived from a WHO grade I meningioma, and is the only such immortal cell line we have identified out of 58 primary cultures of benign meningiomas collected. KCI-MENG1-LP is a heterogeneous cell population comprised of two cellular morphologies, while at high passage (KCI-MENG1-HP), only one of the two cell types remains (likely due to selection during the culturing process). All cell lines derived from KCI-MENG1 retained the expression of the meningioma diagnostic markers EMA and vimentin, which were weakly and strongly stained in the patient tumor specimen, respectively. EMA expression varied between the two cell types (very strong in the round cells, weak in the spindle cells) but both cell types were strongly stained for vimentin. The smaller, round cell phenotype has stronger Ki-67 staining, and maintains the expression of EMA and vimentin through a high number of passages. The difference in the growth kinetics of the cells at low vs. high passages and the marked increase of telomerase activity seem to be congruent with the shift in population density reflecting the loss of the spindle-shaped cells. Furthermore, the higher proportion of cells with the aberrant karyotype and higher magnitude of amplification of cancer-related genes identified by aCGH, particularly in chromosomes 1, 9, 12, 15, 17, and 20 which are known chromosomal gains in atypical and malignant meningioma [25], suggest that, although initially a minor subclone in the original meningioma tumor, the round cells with the high proliferative activity (KCI-MENG1-HP) are likely to be the tumorigenic cells responsible for the tumor development and growth in the patient. This is also supported by our development of subcutaneous tumors from both KCI-MENG1-LP and KCI-MENG1-HP cells in immunocompromised mice, and the high proliferative activity of the tumors generated from subdural implantation. Previous studies of xenografts generated from the malignant meningioma cell lines IOMM-Lee and CH-157, and patient-derived cells demonstrated that meningioma cells with a complex karyotype, such as our KCI-MENG1-HP, more consistently generated tumors after subcutaneous implantation than those with a simple karyotype [44]. This likely manifested as an in vivo clonal selection and accounts for our finding that the KCI-MENG1-LPSX-CL cells isolated from the low passage subcutaneous tumor were a homogeneous population that resembled the KCI-MENG1-HP cells. Interestingly, absence of PR immunostaining in the more aggressive high passages cells is in keeping with the known association of loss of PR expression, cumulative karyotype abnormalities, and aggressive clinical behavior of progressive or recurrent meningiomas [45].

In addition to vimentin, we found expression of N-cadherin in both round and spindle-shaped cell types of the low passage cells, both of which are considered markers of mesenchymal phenotype associated with the invasive properties of some cancer types [46]. N-cadherin expression was previously described in a subset of WHO grade I meningiomas [47] and co-expression of vimentin and N-cadherin was also found in drug-resistant lung cancer [48] and pancreatic carcinoma [49]. Diminished E-cadherin expression is commonly found in all grades of meningioma [50], and in our study, the original patient tumor, mouse xenograft tumors, and all cell lines were negative for E-cadherin immunostaining (see Figure 4) despite the genomic amplification of the *CDH1* gene (which encodes E-cadherin) identified in the cells (see Additional file 2: Table S2), implying that the mesenchymal phenotype predominates.

The property that cells from all cancer types have in common is the ability to propagate indefinitely. Cancer cells typically achieve this by expressing telomerase, which is absent in senescent, differentiated cells [51]. The telomerase activity and resulting telomere dysfunction contributes to genomic instability [52] and can lead to the generation of polyploid cells and enhance the tumorigenicity of those cells [28], which corresponds to our findings of the robust telomerase activity and near-triploid karyotype of the round phenotype KCI-MENG1-HP cells. In meningiomas, telomerase activity tends to correlate with WHO grade and is observed in up to 95% in anaplastic meningiomas [14, 53–55], though rarely found in benign meningiomas [15]. Telomerase inhibitors are currently under development, but not for meningiomas or other brain tumors [51].

Collectively, despite KCI-MENG1 cells originating from a WHO grade I meningioma, our data suggest that these cells have a genomic complexity and a biological profile that is consistent with recurrent and/or high-grade meningiomas. In a cytogenetic study of recurrent, progressive meningioma, Al-Mefty et al. described similar findings [26]. When they assayed meningioma specimens from the initial low-grade tumors from patients that later developed high-grade tumors, they found cells with the aberrant high-grade karyotype in the benign tumors. In a similar line of research, cytogenetic heterogeneity was identified in 33.4% of meningiomas, and it was found that tumor progression and recurrence was predicted by the most advanced clone even if present in lesser abundance [56]. Although the patient from which KCI-MENG1 cells are derived currently has no clinical or radiographic evidence of tumor recurrence 4 years following surgery, the isolation of the near-triploid cells, including a gain of chromosome 1q (known to correlate with shorter progression-free survival in atypical meningioma [27] ), and with high telomerase activity (usually associated with high-grade meningioma [53–55] and demonstrated in meningiomas undergoing malignant progression [57]) is worrisome and suggestive that this patient could be at high risk to have a progressive recurrence. This patient population is one we hope to serve with translational studies of meningioma tumor biology and disease progression utilizing our KCI-MENG1 in vitro and in vivo models, and as such will facilitate further development of novel therapies to improve treatment options for all grades of meningiomas.

## Conclusions

Although derived from a typically good prognosis benign meningioma specimen, the newly-established spontaneously immortal KCI-MENG1 meningioma cell line can be utilized to generate xenograft tumor models with low- or high-grade features, dependent on the cell passage number (likely due to the relative abundance of the near-triploid, likely poor prognosis, cells that were present as a small proportion in the original tumor). These human meningioma mouse xenograft models will provide biologically relevant platforms from which to investigate differences in low- vs. high-grade meningioma tumor biology and disease progression as well as to develop novel therapies to improve treatment options for poor prognosis or recurrent meningiomas.

## Additional files

Additional file 1: Table S1. Cancer Gene List used for aCGH data filtering. Additional file 2: Table S2. aCGH data of Low- and High-Passage KCI-MENG1 Cells.

## Abbreviations

aCGH: array comparative genomic hybridization
EDTA: ethylenediaminetetraacetic acid
EMA: epithelial membrane antigen
H&E: hematoxylin and eosin
hTERT: telomerase catalytic subunit
IHC: immunohistochemistry
MRI: magnetic resonance imaging
PBS: phosphate-buffered saline
PR: progesterone receptor
WHO: World Health Organization
