# Supplementary material for: Development of patient-derived xenograft models from a spontaneously immortal low-grade meningioma cell line, KCI-MENG1
Source: J Transl Med. 2015 Jul 15;13:227. doi: 10.1186/s12967-015-0596-8 (PMC4501087; doi:10.1186/s12967-015-0596-8)
Supplement: Additional file 1: Table S1. — Cancer Gene List used for aCGH data filtering. [file 12967_2015_596_MOESM1_ESM.pdf]

Additional Table 1: Cancer Gene List used for aCGH data filtering

ABI1	BRCA1	CDX2	ELF4	FLCN	HOXD13	MAFB	MYCN	PDCD1LG2	RBM15	SS18L1	TPM3
ABL1	BRCA2	CEBPA	ELK4	FLI1	HRAS	MALAT1	MYD88	PDE4DIP	RECQL4	SSX1	TPM4
ABL2	BRD3	CEP1	ELL	FLT3	HSPCA	MALT1	MYH11	PDGFB	REL	SSX2	TPR
ACSL3	BRD4	CEP76	ELN	FNBP1	HSPCB	MAML2	MYH9	PDGFRA	RET	SSX4	TPX2
AFF4	BRIP1	CHCHD7	EML4	FOXA1	IDH1	MAP2K1	MYOCD	PDGFRB	RHEB	STAG2	TRA
AKAP9	BTG1	CHD8	EP300	FOXL2	IDH2	MAP2K2	MYST4	PER1	RHOA	STAT3	TRAF7
AKT1	BUB1B	CHEK2	EPS15	FOXO1A	IGH	MAP2K4	NACA	PHF6	RNF213	STAT5B	TRB
AKT2	C12orf9	CHIC2	ERBB2	FOXO3A	IGK	MAP4K3	NBS1	PHOX2B	RNF43	STIL	TRD
ALDH2	C15orf21	CHN1	ERC1	FOXP1	IGL	MAX	NCKIPSD	PICALM	ROS1	STK11	TRIM23
ALK	C15orf55	CIC	ERCC2	FSTL3	IKZF1	MBD1	NCOA1	PIK3CA	RPL10	STL	TRIM27
ALPK2	C15orf65	CIITA	ERCC3	FUBP1	IL2	MDM2	NCOA2	PIK3R1	RPL22	STX2	TRIM33
AMER1	C16orf75	CLP1	ERCC4	FUS	IL21R	MDM4	NCOA4	PIM1	RPL5	SUFU	TRIP11
APC	C2orf44	CLTC	ERCC5	FVT1	IL6ST	MDS1	NDRG1	PLA2G10	RPL5	SUZ12	TRRAP
AR	CACNA1D	CLTCL1	ERG	GAS7	IL7R	MDS2	NF1	PLAG1	RPN1	SYK	TSC1
ARHGAP26	CALR	CMKOR1	ETV1	GATA1	IRF4	MECT1	NF2	PML	RUNDC2A	TAF15	TSC2
ARHGEF12	CAMTA1	CNOT3	ETV4	GATA2	IRF6	MED12	NFE2L2	PMS1	RUNX1	TAL1	TSHR
ARHH	CANT1	COL1A1	ETV5	GATA3	ITK	MEN1	NFIB	PMS2	RUNXBP2	TAL2	TTL
ARID1A	CARD11	COPEB	ETV6	GMPS	JAK1	MET	NFKB2	PMX1	RXRA	TAP1	U2AF1
ARID2	CARS	COX6C	EVI1	GNA11	JAK2	MGA	NIN	PNUTL1	SBDS	TBL1XR1	UBR5
ARNT	CASC5	CREB1	EWSR1	GNAQ	JAK3	MITF	NKX2-1	POT1	SDC4	TCEA1	USP6
ASPSCR1	CASP8	CREB3L1	EXT1	GNAS	JAZF1	MKL1	NONO	POU2AF1	SDHAF2	TCF1	VHL
ASXL1	CBFA2T1	CREB3L2	EXT2	GOLGA5	JUN	MLF1	NOTCH1	POU5F1	SDHB	TCF12	VTI1A
ATF1	CBFA2T3	CREBBP	EZH1	GOPC	KCNJ5	MLH1	NOTCH2	PPARG	SDHC	TCF3	WAS
ATIC	CBFB	CRLF2	EZH2	GPC3	KDM5A	MLL	NPM1	PPP2R1A	SDHD	TCF7L2	WHSC1
ATM	CBL	CRTC3	EZR	GPHN	KDM5C	MLL2	NR4A3	PRCC	SEPT6	TCL1A	WHSC1L1
ATP1A1	CBLB	CSF3R	FACL6	GRLF1	KDM6A	MLL3	NRAS	PRDM1	SET	TCL6	WIF1
ATP2B3	CBLC	CTNNB1	FAM22A	H3F3A	KDR	MLLT1	NSD1	PRDM16	SETBP1	TERT	WRN
ATRX	CCDC6	CYLD	FAM22B	H3F3B	KIAA1549	MLLT10	NT5C2	PRF1	SETD2	TET1	WT1
AURKA	CCNB1IP1	D10S170	FAM46C	HERPUD1	KIF5B	MLLT11	NTRK1	PRKAR1A	SETDB1	TET2	WWTR1
AXIN1	CCND1	DAXX	FANCA	HEY1	KIT	MLLT2	NTRK3	PSIP2	SF3B1	TFE3	XPA
BAP1	CCND2	DDB2	FANCC	HIP1	KLF4	MLLT3	NUMA1	PTCH	SFPQ	TFEB	XPC
BCL10	CCND3	DDIT3	FANCD2	HIST1H3B	KLK2	MLLT4	NUP214	PTCH1	SFRS3	TFG	XPO1
BCL11A	CCNE1	DDR2	FANCE	HIST1H4E	KRAS	MLLT6	NUP98	PTEN	SH2B3	TFPT	YWHAE
BCL11B	CD1D	DDX10	FANCF	HIST1H4I	KTN1	MLLT7	OLIG2	PTPN11	SH3GL1	TFRC	ZNF145
BCL2	CD274	DDX5	FANCG	HLA-B	LAF4	MN1	OMD	PTPRC	SLC34A2	THRAP3	ZNF198
BCL3	CD74	DDX6	FBXO11	HLF	LASP1	MPL	P2RY8	QKI	SLC45A3	TIF1	ZNF278
BCL5	CD79A	DEK	FBXW7	HLXB9	LCK	MSF	PAFAH1B2	RABEP1	SMARCA4	TLX1	ZNF331
BCL6	CD79B	DICER1	FCGR2B	HMGA1	LCP1	MSH2	PALB2	RAC1	SMARCB1	TLX3	ZNF384
BCL7A	CDC73	DNER	FCRL4	HMGA2	LHFP	MSH6	PAX3	RAD21	SMARCE1	TMPRSS2	ZNF521
BCL9	CDH1	DNM2	FEV	HNRNPA2B1	LIFR	MSI2	PAX5	RAD21	SMO	TNF	ZNF750
BCLAF1	CDH11	DNMT3A	FGFR1	HOOK3	LMO1	MSN	PAX7	RAD51L1	SOCS1	TNFAIP3	ZNF9
BCOR	CDK12	DUX4	FGFR1OP	HOXA11	LMO2	MTCP1	PAX8	RAF1	SOS1	TNFRSF14	ZRANB3
BCR	CDK4	EBF1	FGFR2	HOXA13	LPP	MUC1	PBRM1	RALGDS	SOX2	TNFRSF17	ZRSR2
BIRC3	CDK6	ECT2L	FGFR3	HOXA9	LRIG3	MUTYH	PBX1	RANBP17	SPECC1	TNFRSF6	
BLM	CDKN2A	EGFR	FH	HOXC11	LYL1	MYB	PCBP1	RAP1GDS1	SRGAP3	TOP1	
BMPR1A	CDKN2AIP	EIF4A2	FHIT	HOXC13	MADH4	MYC	PCM1	RARA	SRSF2	TP53	
BRAF	CDKN2C	ELF3	FIP1L1	HOXD11	MAF	MYCL1	PCSK7	RB1	SS18	TP53BP1	
