# Supplementary material for: Development of patient-derived xenograft models from a spontaneously immortal low-grade meningioma cell line, KCI-MENG1
Source: J Transl Med. 2015 Jul 15;13:227. doi: 10.1186/s12967-015-0596-8 (PMC4501087; doi:10.1186/s12967-015-0596-8)
Supplement: Additional file 2: Table S2. — aCGH data of Low- and High-Passage KCI-MENG1 Cells. [file 12967_2015_596_MOESM2_ESM.pdf]

ADDITIONAL TABLE 2: aCGH data of Low- and High-Passage KCI-MENG1 Cells

Chr	CytoBand	GeneID	Gene name	Amplification P6	Deletion P6	Amplification P86	Deletion P86
chr01	q21.1 - q44	ABL2	v-abl Abelson murine leukemia viral oncogene homolog 2 (arg, Abelson-related gene)			0.890879	
chr01	q21.1 - q44	ARNT	aryl hydrocarbon receptor nuclear translocator			0.890879	
chr01	q21.1 - q44	BCL9	B-cell CLL/lymphoma 9			0.890879	
chr01	q21.1 - q44	CDC73	cell division cycle 73, Paf1/RNA polymerase II complex component, homolog (S. cerevisiae)			0.890879	
chr01	q21.1 - q44	ELK4	ELK4, ETS-domain protein (SRF accessory protein 1)			0.890879	
chr01	q21.1 - q44	FCGR2B	Fc fragment of IgG, low affinity IIb, receptor (CD32) Fc fragment of IgG, low affinity IIc, receptor for (CD32)			0.890879	
chr01	q21.1 - q44	FCRL4	Fc receptor-like 4			0.890879	
chr01	q21.1 - q44	FH	fumarate hydratase			0.890879	
chr01	q21.1 - q44	H3F3A	H3 histone, family 3B (H3.3B) H3 histone, family 3A pseudogene			0.890879	
chr01	q21.1 - q44	MDM4	Mdm4 p53 binding protein homolog (mouse)			0.890879	
chr01	q21.1 - q44	MLLT11	myeloid/lymphoid or mixed-lineage leukemia (trithorax homolog, Drosophila) translocated to, 11			0.890879	
chr01	q21.1 - q44	MUC1	mucin 1, cell surface associated			0.890879	
chr01	q21.1 - q44	NTRK1	neurotrophic tyrosine kinase, receptor, type 1			0.890879	
chr01	q21.1 - q44	PBX1	pre-B-cell leukemia homeobox 1			0.890879	
chr01	q21.1 - q44	PDE4DIP	programmed cell death 1 ligand 2			0.890879	
chr01	q21.1 - q44	PRCC	papillary renal cell carcinoma (translocation-associated)			0.890879	
chr01	q21.1 - q44	PRRX1	paired related homeobox 1			0.890879	
chr01	q21.1 - q44	SDHC	succinate dehydrogenase complex, subunit C, integral membrane protein, 15kDa			0.890879	
chr01	q21.1 - q44	SLC45A3	splicing factor proline/glutamine-rich (polypyrimidine tract binding protein associated)			0.890879	
chr01	q21.1 - q44	TPM3	tropomyosin 3			0.890879	
chr01	q21.1 - q44	TPR	translocated promoter region (to activated MET oncogene)			0.890879	
chr01	p22.1 - p11.2	FAM46C	family with sequence similarity 46, member C			0.817825	
chr01	p22.1 - p11.2	NOTCH2	Notch homolog 2 (Drosophila)			0.817825	
chr01	p22.1 - p11.2	NRAS	neuroblastoma RAS viral (v-ras) oncogene homolog			0.817825	
chr01	p22.1 - p11.2	TRIM33	tripartite motif-containing 33			0.817825	
chr01	p36.33 - p11.2	ARID1A	AT rich interactive domain 1A (SWI-like)			0.598554	
chr01	p36.33 - p11.2	BCL10	B-cell CLL/lymphoma 10 hypothetical LOC646626			0.598554	
chr01	p36.33 - p11.2	CAMTA1	calmodulin binding transcription activator 1			0.598554	
chr01	p36.33 - p11.2	CDKN2C	cyclin-dependent kinase inhibitor 2C (p18, inhibits CDK4)			0.598554	
chr01	p36.33 - p11.2	EPS15	epidermal growth factor receptor pathway substrate 15			0.598554	
chr01	p36.33 - p11.2	FAM46C	family with sequence similarity 46, member C			0.598554	
chr01	p36.33 - p11.2	FUBP1	far upstream element (FUSE) binding protein 1			0.598554	
chr01	p36.33 - p11.2	JAK1	Janus kinase 1			0.598554	
chr01	p36.33 - p11.2	JUN	jun oncogene			0.598554	
chr01	p36.33 - p11.2	LCK	lymphocyte-specific protein tyrosine kinase			0.598554	
chr01	p36.33 - p11.2	MDS2	myelodysplastic syndrome 2 translocation associated			0.598554	
chr01	p36.33 - p11.2	MPL	myeloproliferative leukemia virus oncogene			0.598554	

ADDITIONAL TABLE 2: aCGH data of Low- and High-Passage KCI-MENG1 Cells

Chr	CytoBand	GeneID	Gene name	Amplification P6	Deletion P6	Amplification P86	Deletion P86
chr01	p36.33 - p11.2	MUTYH	mutY homolog (E. coli)			0.598554	
chr01	p36.33 - p11.2	MYCL1	v-myc myelocytomatosis viral oncogene homolog 1, lung carcinoma derived (avian)			0.598554	
chr01	p36.33 - p11.2	NOTCH2	Notch homolog 2 (Drosophila)			0.598554	
chr01	p36.33 - p11.2	NRAS	neuroblastoma RAS viral (v-ras) oncogene homolog			0.598554	
chr01	p36.33 - p11.2	PAX7	paired box 7			0.598554	
chr01	p36.33 - p11.2	PRDM16	PR domain containing 16			0.598554	
chr01	p36.33 - p11.2	RBM15	RNA binding motif protein 15			0.598554	
chr01	p36.33 - p11.2	RBM15	RNA binding motif protein 15			0.598554	
chr01	p36.33 - p11.2	RPL22	ribosomal protein L22 pseudogene 11 ribosomal protein L22			0.598554	
chr01	p36.33 - p11.2	SDHB	succinate dehydrogenase complex, subunit B, iron sulfur (Ip)			0.598554	
chr01	p36.33 - p11.2	SFPQ	splicing factor proline/glutamine-rich (polypyrimidine tract binding protein associated)			0.598554	
chr01	p36.33 - p11.2	STIL	SCL/TAL1 interrupting locus			0.598554	
chr01	p36.33 - p11.2	TAL1	T-cell acute lymphocytic leukemia 1			0.598554	
chr01	p36.33 - p11.2	THRAP3	thyroid hormone receptor associated protein 3			0.598554	
chr01	p36.33 - p11.2	TNFRSF14	tumor necrosis factor receptor superfamily, member 14 (herpesvirus entry mediator)			0.598554	
chr01	p36.33 - p11.2	TRIM33	tripartite motif-containing 33			0.598554	
chr01	p36.33 - p32.3	ARID1A	AT rich interactive domain 1A (SWI-like)			0.51241	
chr01	p36.33 - p32.3	CAMTA1	calmodulin binding transcription activator 1			0.51241	
chr01	p36.33 - p32.3	CDKN2C	cyclin-dependent kinase inhibitor 2C (p18, inhibits CDK4)			0.51241	
chr01	p36.33 - p32.3	LCK	lymphocyte-specific protein tyrosine kinase			0.51241	
chr01	p36.33 - p32.3	MDS2	myelodysplastic syndrome 2 translocation associated			0.51241	
chr01	p36.33 - p32.3	MPL	myeloproliferative leukemia virus oncogene			0.51241	
chr01	p36.33 - p32.3	MUTYH	mutY homolog (E. coli)			0.51241	
chr01	p36.33 - p32.3	MYCL1	v-myc myelocytomatosis viral oncogene homolog 1, lung carcinoma derived (avian)			0.51241	
chr01	p36.33 - p32.3	PAX7	paired box 7			0.51241	
chr01	p36.33 - p32.3	PRDM16	PR domain containing 16			0.51241	
chr01	p36.33 - p32.3	RPL22	ribosomal protein L22 pseudogene 11 ribosomal protein L22			0.51241	
chr01	p36.33 - p32.3	SDHB	succinate dehydrogenase complex, subunit B, iron sulfur (Ip)			0.51241	
chr01	p36.33 - p32.3	SFPQ	splicing factor proline/glutamine-rich (polypyrimidine tract binding protein associated)			0.51241	
chr01	p36.33 - p32.3	STIL	SCL/TAL1 interrupting locus			0.51241	
chr01	p36.33 - p32.3	TAL1	T-cell acute lymphocytic leukemia 1			0.51241	
chr01	p36.33 - p32.3	THRAP3	thyroid hormone receptor associated protein 3			0.51241	
chr01	p36.33 - p32.3	TNFRSF14	tumor necrosis factor receptor superfamily, member 14 (herpesvirus entry mediator)			0.51241	
chr02	p22.1 - p16.1	EML4	echinoderm microtubule associated protein like 4	0.324536		0.986305	
chr02	p22.1 - p16.1	FBXO11	F-box protein 11	0.324536		0.986305	
chr02	p22.1 - p16.1	MSH2	mutS homolog 2, colon cancer, nonpolyposis type 1 (E. coli)	0.324536		0.986305	
chr02	p22.1 - p16.1	MSH6	mutS homolog 6 (E. coli)	0.324536		0.986305	

ADDITIONAL TABLE 2: aCGH data of Low- and High-Passage KCI-MENG1 Cells

Chr	CytoBand	GeneID	Gene name	Amplification P6	Deletion P6	Amplification P86	Deletion P86
chr02	q11.1 - q14.3	AFF3	AF4/FMR2 family, member 3			0.898312	
chr02	q11.1 - q14.3	ERCC3	excision repair cross-complementing rodent repair deficiency, complementation group 3 (xeroderma pigmentosum group B complementing)			0.898312	
chr02	q11.1 - q14.3	PAX8	paired box 8			0.898312	
chr02	q11.1 - q14.3	TTL	tubulin tyrosine ligase			0.898312	
chr02	p22.2 - p11.1	BCL11A	B-cell CLL/lymphoma 11A (zinc finger protein)			0.70192	
chr02	p22.2 - p11.1	EML4	echinoderm microtubule associated protein like 4			0.70192	
chr02	p22.2 - p11.1	FBXO11	F-box protein 11			0.70192	
chr02	p22.2 - p11.1	MSH2	mutS homolog 2, colon cancer, nonpolyposis type 1 (E. coli)			0.70192	
chr02	p22.2 - p11.1	MSH6	mutS homolog 6 (E. coli)			0.70192	
chr02	p22.2 - p11.1	REL	v-rel reticuloendotheliosis viral oncogene homolog (avian)			0.70192	
chr02	p22.2 - p11.1	XPO1	exportin 1 (CRM1 homolog, yeast)			0.70192	
chr02	p16.1 - p11.2	BCL11A	B-cell CLL/lymphoma 11A (zinc finger protein)			0.533694	
chr02	p16.1 - p11.2	REL	v-rel reticuloendotheliosis viral oncogene homolog (avian)			0.533694	
chr02	p16.1 - p11.2	XPO1	exportin 1 (CRM1 homolog, yeast)			0.533694	
chr03	q26.1 - q26.2	MECOM	ecotropic viral integration site 1	0.702415		1.47986	
chr03	q11.1 - q26.2	CBLB	Cas-Br-M (murine) ecotropic retroviral transforming sequence b			0.933628	
chr03	q11.1 - q26.2	CNBP	CCHC-type zinc finger, nucleic acid binding protein			0.933628	
chr03	q11.1 - q26.2	FOXL2	forkhead box L2			0.933628	
chr03	q11.1 - q26.2	GATA2	GATA binding protein 2			0.933628	
chr03	q11.1 - q26.2	GMPS	guanine monphosphate synthetase			0.933628	
chr03	q11.1 - q26.2	MECOM	ecotropic viral integration site 1			0.933628	
chr03	q11.1 - q26.2	MLF1	myeloid leukemia factor 1			0.933628	
chr03	q11.1 - q26.2	RPN1	ribophorin I			0.933628	
chr03	q11.1 - q26.2	TFG	TRK-fused gene			0.933628	
chr03	q11.1 - q26.2	WWTR1	WW domain containing transcription regulator 1			0.933628	
chr03	p26.3 - p11.1	BAP1	BRCA1 associated protein-1 (ubiquitin carboxy-terminal hydrolase)			0.588208	
chr03	p26.3 - p11.1	CTNNB1	catenin (cadherin-associated protein), beta 1, 88kDa			0.588208	
chr03	p26.3 - p11.1	FANCD2	Fanconi anemia, complementation group D2			0.588208	
chr03	p26.3 - p11.1	FHIT	fragile histidine triad gene			0.588208	
chr03	p26.3 - p11.1	FOXP1	forkhead box P1			0.588208	
chr03	p26.3 - p11.1	MITF	microphthalmia-associated transcription factor			0.588208	
chr03	p26.3 - p11.1	MLH1	mutL homolog 1, colon cancer, nonpolyposis type 2 (E. coli)			0.588208	
chr03	p26.3 - p11.1	MYD88	myeloid differentiation primary response gene (88)			0.588208	
chr03	p26.3 - p11.1	NCKIPSD	nascent polypeptide-associated complex alpha subunit			0.588208	
chr03	p26.3 - p11.1	PBRM1	polybromo 1			0.588208	
chr03	p26.3 - p11.1	PPARG	peroxisome proliferator-activated receptor gamma			0.588208	
chr03	p26.3 - p11.1	RAF1	v-raf-1 murine leukemia viral oncogene homolog 1			0.588208	
chr03	p26.3 - p11.1	SETD2	SET domain containing 2			0.588208	
chr03	p26.3 - p11.1	SRGAP3	SLIT-ROBO Rho GTPase activating protein 3			0.588208	
chr03	p26.3 - p11.1	VHL	von Hippel-Lindau tumor suppressor			0.588208	
chr03	p26.3 - p11.1	XPC	xeroderma pigmentosum, complementation group C			0.588208	

ADDITIONAL TABLE 2: aCGH data of Low- and High-Passage KCI-MENG1 Cells

Chr	CytoBand	GeneID	Gene name	Amplification P6	Deletion P6	Amplification P86	Deletion P86
chr03	p14.2	FHIT	fragile histidine triad gene		-0.614211		-0.501957
chr03	q12.2 - q26.2	CBLB	Cas-Br-M (murine) ecotropic retroviral transforming sequence b	0.308483			
chr03	q12.2 - q26.2	CNBP	CCHC-type zinc finger, nucleic acid binding protein	0.308483			
chr03	q12.2 - q26.2	FOXL2	forkhead box L2	0.308483			
chr03	q12.2 - q26.2	GATA2	GATA binding protein 2	0.308483			
chr03	q12.2 - q26.2	GMPS	guanine monphosphate synthetase	0.308483			
chr03	q12.2 - q26.2	MECOM	ecotropic viral integration site 1	0.308483			
chr03	q12.2 - q26.2	MLF1	myeloid leukemia factor 1	0.308483			
chr03	q12.2 - q26.2	RPN1	ribophorin I	0.308483			
chr03	q12.2 - q26.2	TFG	TRK-fused gene	0.308483			
chr03	q12.2 - q26.2	WWTR1	WW domain containing transcription regulator 1	0.308483			
chr05	p15.33 - p11	IL7R	interleukin 7 receptor	1.213931		2.050158	
chr05	p15.33 - p11	LIFR	leukemia inhibitory factor receptor alpha	1.213931		2.050158	
chr05	q33.2 - q35.2	EBF1	early B-cell factor 1			0.905554	
chr05	q33.2 - q35.2	ITK	IL2-inducible T-cell kinase			0.905554	
chr05	q33.2 - q35.2	NPM1	nucleophosmin 1 (nucleolar phosphoprotein B23, numatrin) pseudogene 21 hypothetical LOC100131044			0.905554	
chr05	q33.2 - q35.2	RANBP17	ral guanine nucleotide dissociation stimulator			0.905554	
chr05	q33.2 - q35.2	TLX3	T-cell leukemia homeobox 3			0.905554	
chr05	q11.1 - q35.3	ACSL6	acyl-CoA synthetase long-chain family member 6			0.588956	
chr05	q11.1 - q35.3	AFF4	AF4/FMR2 family, member 4			0.588956	
chr05	q11.1 - q35.3	APC	adenomatous polyposis coli			0.588956	
chr05	q11.1 - q35.3	ARHGAP26	Rho GTPase activating protein 26			0.588956	
chr05	q11.1 - q35.3	CD74	CD74 molecule, major histocompatibility complex, class II invariant chain			0.588956	
chr05	q11.1 - q35.3	EBF1	early B-cell factor 1			0.588956	
chr05	q11.1 - q35.3	IL6ST	interleukin 6 signal transducer (gp130, oncostatin M receptor)			0.588956	
chr05	q11.1 - q35.3	ITK	IL2-inducible T-cell kinase			0.588956	
chr05	q11.1 - q35.3	NPM1	nucleophosmin 1 (nucleolar phosphoprotein B23, numatrin) pseudogene 21 hypothetical LOC100131044			0.588956	
chr05	q11.1 - q35.3	NSD1	nuclear receptor binding SET domain protein 1			0.588956	
chr05	q11.1 - q35.3	PDGFRB	platelet-derived growth factor receptor, beta polypeptide			0.588956	
chr05	q11.1 - q35.3	PIK3R1	phosphoinositide-3-kinase, regulatory subunit 1 (alpha)			0.588956	
chr05	q11.1 - q35.3	RANBP17	ral guanine nucleotide dissociation stimulator			0.588956	
chr05	q11.1 - q35.3	TLX3	T-cell leukemia homeobox 3			0.588956	
chr05	q21.3 - q32	ACSL6	acyl-CoA synthetase long-chain family member 6			0.521088	
chr05	q21.3 - q32	AFF4	AF4/FMR2 family, member 4			0.521088	
chr05	q21.3 - q32	APC	adenomatous polyposis coli			0.521088	
chr05	q21.3 - q32	ARHGAP26	Rho GTPase activating protein 26			0.521088	
chr06	q22.1 - q22.31	GOPC	golgi associated PDZ and coiled-coil motif containing			0.585527	
chr06	q22.1 - q22.31	ROS1	c-ros oncogene 1 , receptor tyrosine kinase			0.585527	
chr06	p25.3 - p11.1	CCND3	cyclin D3			0.53958	
chr06	p25.3 - p11.1	DAXX	death-domain associated protein			0.53958	

ADDITIONAL TABLE 2: aCGH data of Low- and High-Passage KCI-MENG1 Cells

Chr	CytoBand	GeneID	Gene name	Amplification P6	Deletion P6	Amplification P86	Deletion P86
chr06	p25.3 - p11.1	DEK	DEK oncogene			0.53958	
chr06	p25.3 - p11.1	FANCE	Fanconi anemia, complementation group E			0.53958	
chr06	p25.3 - p11.1	HIST1H4I	histone cluster 1, H4l histone cluster 4, H4 histone cluster 1, H4h histone cluster 1, H4j histone cluster 1, H4i histone cluster 1, H4d histone cluster 1, H4c histone cluster 1, H4f histone cluster 1, H4e histone cluster 1, H4b histone cluster 1, H4a histone cluster 2, H4a histone cluster 2, H4b histone cluster 1, H4k			0.53958	
chr06	p25.3 - p11.1	HMGA1	hypothetical LOC100130009 high mobility group AT-hook 1			0.53958	
chr06	p25.3 - p11.1	HSP90AB1	heat shock protein 90kDa alpha (cytosolic), class B member 1			0.53958	
chr06	p25.3 - p11.1	IRF4	interferon regulatory factor 4			0.53958	
chr06	p25.3 - p11.1	PIM1	pim-1 oncogene			0.53958	
chr06	p25.3 - p11.1	POU5F1	POU class 5 homeobox 1			0.53958	
chr06	p25.3 - p11.1	SRSF3	SLIT-ROBO Rho GTPase activating protein 3			0.53958	
chr06	p25.3 - p11.1	TFEB	transcription factor EB			0.53958	
chr06	p25.3 - p11.1	TRIM27	tripartite motif-containing 27			0.53958	
chr06	q27	FGFR1OP	Fc receptor-like 4			0.473619	
chr07	q21.11 - q22.1	AKAP9	A kinase (PRKA) anchor protein (yotiao) 9			0.987225	
chr07	q21.11 - q22.1	CDK6	cyclin-dependent kinase 6			0.987225	
chr07	p22.3 - p11.1	CARD11	caspase recruitment domain family, member 11			0.913181	
chr07	p22.3 - p11.1	EGFR	epidermal growth factor receptor (erythroblastic leukemia viral (v-erb-b) oncogene homolog, avian)			0.913181	
chr07	p22.3 - p11.1	ETV1	ets variant 1			0.913181	
chr07	p22.3 - p11.1	HNRNPA2B1	heterogeneous nuclear ribonucleoprotein A2/B1			0.913181	
chr07	p22.3 - p11.1	HOXA11	homeobox A11			0.913181	
chr07	p22.3 - p11.1	HOXA13	homeobox A13			0.913181	
chr07	p22.3 - p11.1	HOXA9	homeobox A9			0.913181	
chr07	p22.3 - p11.1	IKZF1	IKAROS family zinc finger 1 (Ikaros)			0.913181	
chr07	p22.3 - p11.1	JAZF1	JAZF zinc finger 1			0.913181	
chr07	p22.3 - p11.1	PMS2	PMS2 postmeiotic segregation increased 2 (S. cerevisiae)			0.913181	
chr07	q33 - q34	BRAF	v-raf murine sarcoma viral oncogene homolog B1			0.887822	
chr07	q33 - q34	CREB3L2	cAMP responsive element binding protein 3-like 2			0.887822	
chr07	q33 - q34	KIAA1549	KIAA1549			0.887822	
chr07	q33 - q34	TRIM24	tripartite motif-containing 24			0.887822	
chr07	q11.1 - q36.3	AKAP9	A kinase (PRKA) anchor protein (yotiao) 9			0.69742	
chr07	q11.1 - q36.3	BRAF	v-raf murine sarcoma viral oncogene homolog B1			0.69742	
chr07	q11.1 - q36.3	CDK6	cyclin-dependent kinase 6			0.69742	
chr07	q11.1 - q36.3	CREB3L2	cAMP responsive element binding protein 3-like 2			0.69742	
chr07	q11.1 - q36.3	ELN	elastin			0.69742	
chr07	q11.1 - q36.3	EZH2	enhancer of zeste homolog 2 (Drosophila)			0.69742	
chr07	q11.1 - q36.3	HIP1	huntingtin interacting protein 1			0.69742	
chr07	q11.1 - q36.3	KIAA1549	KIAA1549			0.69742	
chr07	q11.1 - q36.3	MET	met proto-oncogene (hepatocyte growth factor receptor)			0.69742	
chr07	q11.1 - q36.3	MLL3	myeloid/lymphoid or mixed-lineage leukemia 3			0.69742	

ADDITIONAL TABLE 2: aCGH data of Low- and High-Passage KCI-MENG1 Cells

Chr	CytoBand	GeneID	Gene name	Amplification P6	Deletion P6	Amplification P86	Deletion P86
chr07	q11.1 - q36.3	MNX1	motor neuron and pancreas homeobox 1			0.69742	
chr07	q11.1 - q36.3	SBDS	Shwachman-Bodian-Diamond syndrome pseudogene Shwachman-Bodian-Diamond syndrome			0.69742	
chr07	q11.1 - q36.3	SMO	smoothened homolog (Drosophila)			0.69742	
chr07	q11.1 - q36.3	TRIM24	tripartite motif-containing 24			0.69742	
chr07	q22.1 - q33	MET	met proto-oncogene (hepatocyte growth factor receptor)			0.554199	
chr07	q22.1 - q33	SMO	smoothened homolog (Drosophila)			0.554199	
chr07	q34 - q36.3	BRAF	v-raf murine sarcoma viral oncogene homolog B1			0.527353	
chr07	q34 - q36.3	EZH2	enhancer of zeste homolog 2 (Drosophila)			0.527353	
chr07	q34 - q36.3	MLL3	myeloid/lymphoid or mixed-lineage leukemia 3			0.527353	
chr07	q34 - q36.3	MNX1	motor neuron and pancreas homeobox 1			0.527353	
chr07	q11.21 - q11.23	ELN	elastin			0.515086	
chr07	q11.21 - q11.23	SBDS	Shwachman-Bodian-Diamond syndrome pseudogene Shwachman-Bodian-Diamond syndrome			0.515086	
chr08	p23.3 - p12	PCM1	pericentriolar material 1			0.586885	
chr08	p23.3 - p12	WRN	similar to Werner syndrome protein Werner syndrome, RecQ helicase-like			0.586885	
chr08	q23.2 - q24.3	EXT1	exostoses (multiple) 1			0.454983	
chr08	q23.2 - q24.3	MYC	v-myc myelocytomatosis viral oncogene homolog (avian)			0.454983	
chr08	q23.2 - q24.3	NDRG1	N-myc downstream regulated 1			0.454983	
chr08	q23.2 - q24.3	RECQL4	RecQ protein-like 4			0.454983	
chr09	q33.1 - q34.3	ABL1	c-abl oncogene 1, receptor tyrosine kinase			0.870693	
chr09	q33.1 - q34.3	BRD3	bromodomain containing 3			0.870693	
chr09	q33.1 - q34.3	FNBP1	formin binding protein 1			0.870693	
chr09	q33.1 - q34.3	NOTCH1	Notch homolog 1, translocation-associated (Drosophila)			0.870693	
chr09	q33.1 - q34.3	NUP214	nucleoporin 214kDa			0.870693	
chr09	q33.1 - q34.3	RALGDS	ral guanine nucleotide dissociation stimulator			0.870693	
chr09	q33.1 - q34.3	SET	SET nuclear oncogene similar to SET translocation			0.870693	
chr09	q33.1 - q34.3	TSC1	tuberous sclerosis 1			0.870693	
chr09	p24.3 - p21.3	CD274	CD274 molecule			0.555255	
chr09	p24.3 - p21.3	JAK2	Janus kinase 2			0.555255	
chr09	p24.3 - p21.3	MLLT3	myeloid/lymphoid or mixed-lineage leukemia (trithorax homolog, Drosophila) translocated to, 3			0.555255	
chr09	p24.3 - p21.3	NFIB	nuclear factor I/B			0.555255	
chr09	p24.3 - p21.3	PDCD1LG2	programmed cell death 1 ligand 2			0.555255	
chr09	p24.3 - p21.3	PSIP1	PC4 and SFRS1 interacting protein 1			0.555255	
chr09	q13 - q22.2	GNAQ	guanine nucleotide binding protein (G protein), q polypeptide			0.41749	
chr10	q11.21 - q22.2	MYST4	MYST histone acetyltransferase (monocytic leukemia) 4	0.499694		1.213813	
chr10	q11.21 - q22.2	NCOA4	nuclear receptor coactivator 4	0.499694		1.213813	
chr10	q11.21 - q22.2	PRF1	perforin 1 (pore forming protein)	0.499694		1.213813	
chr10	q11.21 - q22.2	TET1	tet oncogene 1	0.499694		1.213813	
chr10	q25.1 - q25.2	VTI1A	vesicle transport through interaction with t-SNAREs homolog 1A (yeast)			0.844808	

ADDITIONAL TABLE 2: aCGH data of Low- and High-Passage KCI-MENG1 Cells

Chr	CytoBand	GeneID	Gene name	Amplification P6	Deletion P6	Amplification P86	Deletion P86
chr10	q11.21 - q26.3	BMPR1A	bone morphogenetic protein receptor, type IA similar to ALK-3			0.785939	
chr10	q11.21 - q26.3	FAM22A	family with sequence similarity 22, member A			0.785939	
chr10	q11.21 - q26.3	FAS	Fas (TNF receptor superfamily, member 6)			0.785939	
chr10	q11.21 - q26.3	FGFR2	fibroblast growth factor receptor 2			0.785939	
chr10	q11.21 - q26.3	MYST4	MYST histone acetyltransferase (monocytic leukemia) 4			0.785939	
chr10	q11.21 - q26.3	NCOA4	nuclear receptor coactivator 4			0.785939	
chr10	q11.21 - q26.3	NFKB2	nuclear factor of kappa light polypeptide gene enhancer in B-cells 2 (p49/p100)			0.785939	
chr10	q11.21 - q26.3	PRF1	perforin 1 (pore forming protein)			0.785939	
chr10	q11.21 - q26.3	PTEN	phosphatase and tensin homolog phosphatase and tensin homolog pseudogene 1			0.785939	
chr10	q11.21 - q26.3	RET	ret proto-oncogene			0.785939	
chr10	q11.21 - q26.3	SUFU	suppressor of fused homolog (Drosophila)			0.785939	
chr10	q11.21 - q26.3	TCF7L2	transcription factor 7-like 2 (T-cell specific, HMG-box)			0.785939	
chr10	q11.21 - q26.3	TET1	tet oncogene 1			0.785939	
chr10	q11.21 - q26.3	TLX1	T-cell leukemia homeobox 1			0.785939	
chr10	q11.21 - q26.3	VTI1A	vesicle transport through interaction with t-SNAREs homolog 1A (yeast)			0.785939	
chr10	q22.3 - q26.3	BMPR1A	bone morphogenetic protein receptor, type IA similar to ALK-3			0.578304	
chr10	q22.3 - q26.3	FAM22A	family with sequence similarity 22, member A			0.578304	
chr10	q22.3 - q26.3	FAS	Fas (TNF receptor superfamily, member 6)			0.578304	
chr10	q22.3 - q26.3	FGFR2	fibroblast growth factor receptor 2			0.578304	
chr10	q22.3 - q26.3	NFKB2	nuclear factor of kappa light polypeptide gene enhancer in B-cells 2 (p49/p100)			0.578304	
chr10	q22.3 - q26.3	PTEN	phosphatase and tensin homolog phosphatase and tensin homolog pseudogene 1			0.578304	
chr10	q22.3 - q26.3	SUFU	suppressor of fused homolog (Drosophila)			0.578304	
chr10	q22.3 - q26.3	TCF7L2	transcription factor 7-like 2 (T-cell specific, HMG-box)			0.578304	
chr10	q22.3 - q26.3	TLX1	T-cell leukemia homeobox 1			0.578304	
chr10	q22.3 - q26.3	VTI1A	vesicle transport through interaction with t-SNAREs homolog 1A (yeast)			0.578304	
chr10	q11.21 - q22.2	RET	ret proto-oncogene	0.499694			
chr11	q21 - q22.2	BIRC3	baculoviral IAP repeat-containing 3	0.899188		1.654617	
chr11	q14.3 - q22.2	BIRC3	baculoviral IAP repeat-containing 3			1.472601	
chr11	q14.3 - q22.2	MAML2	mastermind-like 2 (Drosophila)			1.472601	
chr11	p11.2 - p11.12	DDB2	damage-specific DNA binding protein 2, 48kDa	0.675591		1.367689	
chr11	q14.1 - q14.2	PICALM	phosphatidylinositol binding clathrin assembly protein			1.233367	
chr11	q13.1 - q13.2	MALAT1	metastasis associated lung adenocarcinoma transcript 1 (non-protein coding)	0.493271		1.166481	
chr11	p15.4 - p15.3	CARS	cysteinyl-tRNA synthetase	0.475881		1.142749	
chr11	p15.4 - p15.3	LMO1	LIM domain only 1 (rhombotin 1)	0.475881		1.142749	
chr11	p15.4 - p15.3	NUP98	nucleoporin 98kDa	0.475881		1.142749	
chr11	q12.1 - q22.2	BIRC3	baculoviral IAP repeat-containing 3			0.982337	

ADDITIONAL TABLE 2: aCGH data of Low- and High-Passage KCI-MENG1 Cells

Chr	CytoBand	GeneID	Gene name	Amplification P6	Deletion P6	Amplification P86	Deletion P86
chr11	q12.1 - q22.2	CCND1	cyclin D1			0.982337	
chr11	q12.1 - q22.2	CLP1	CLP1, cleavage and polyadenylation factor I subunit, homolog (S. cerevisiae)			0.982337	
chr11	q12.1 - q22.2	MALAT1	metastasis associated lung adenocarcinoma transcript 1 (non-protein coding)			0.982337	
chr11	q12.1 - q22.2	MAML2	mastermind-like 2 (Drosophila)			0.982337	
chr11	q12.1 - q22.2	MEN1	multiple endocrine neoplasia I			0.982337	
chr11	q12.1 - q22.2	NUMA1	nuclear mitotic apparatus protein 1			0.982337	
chr11	q12.1 - q22.2	PICALM	phosphatidylinositol binding clathrin assembly protein			0.982337	
chr11	q12.1 - q22.2	SDHAF2	chromosome 11 open reading frame 79			0.982337	
chr11	p15.4 - p15.3	LMO1	LIM domain only 1 (rhombotin 1)			0.914575	
chr11	p15.4 - p11.12	CARS	cysteinyl-tRNA synthetase			0.844095	
chr11	p15.4 - p11.12	CREB3L1	collagen, type I, alpha 1			0.844095	
chr11	p15.4 - p11.12	DDB2	damage-specific DNA binding protein 2, 48kDa			0.844095	
chr11	p15.4 - p11.12	EXT2	exostoses (multiple) 2			0.844095	
chr11	p15.4 - p11.12	FANCF	Fanconi anemia, complementation group F			0.844095	
chr11	p15.4 - p11.12	LMO1	LIM domain only 1 (rhombotin 1)			0.844095	
chr11	p15.4 - p11.12	LMO2	LIM domain only 2 (rhombotin-like 1)			0.844095	
chr11	p15.4 - p11.12	NUP98	nucleoporin 98kDa			0.844095	
chr11	p15.4 - p11.12	WT1	Wilms tumor 1			0.844095	
chr11	q24.3 - q25	FLI1	Friend leukemia virus integration 1			0.578688	
chr11	p12 - p11.2	EXT2	exostoses (multiple) 2			0.557571	
chr11	p15.2 - p14.3	FANCF	Fanconi anemia, complementation group F			0.536336	
chr11	q22.2 - q25	ARHGEF12	Rho guanine nucleotide exchange factor (GEF) 12			0.375304	
chr11	q22.2 - q25	ATM	similar to Serine-protein kinase ATM (Ataxia telangiectasia mutated) (A-T, mutated) ataxia telangiectasia mutated			0.375304	
chr11	q22.2 - q25	BIRC3	baculoviral IAP repeat-containing 3			0.375304	
chr11	q22.2 - q25	CBL	Cas-Br-M (murine) ecotropic retroviral transforming sequence			0.375304	
chr11	q22.2 - q25	DDX10	DEAD (Asp-Glu-Ala-Asp) box polypeptide 10			0.375304	
chr11	q22.2 - q25	DDX6	DEAD (Asp-Glu-Ala-Asp) box polypeptide 6			0.375304	
chr11	q22.2 - q25	FLI1	Friend leukemia virus integration 1			0.375304	
chr11	q22.2 - q25	MLL	myeloid/lymphoid or mixed-lineage leukemia (trithorax homolog, Drosophila)			0.375304	
chr11	q22.2 - q25	PAFAH1B2	platelet-activating factor acetylhydrolase, isoform Ib, subunit 2 (30kDa)			0.375304	
chr11	q22.2 - q25	PCSK7	proprotein convertase subtilisin/kexin type 7 pseudogene proprotein convertase subtilisin/kexin type 7			0.375304	
chr11	q22.2 - q25	POU2AF1	PMS2 postmeiotic segregation increased 2 (S. cerevisiae)			0.375304	
chr11	q22.2 - q25	SDHD	similar to succinate dehydrogenase complex, subunit D, integral membrane protein succinate dehydrogenase complex, subunit D, integral membrane protein			0.375304	
chr11	q22.2 - q25	ZBTB16	zinc finger and BTB domain containing 16			0.375304	
chr11	q14.1- q22.2	BIRC3	baculoviral IAP repeat-containing 3	0.709864			

ADDITIONAL TABLE 2: aCGH data of Low- and High-Passage KCI-MENG1 Cells

Chr	CytoBand	GeneID	Gene name	Amplification P6	Deletion P6	Amplification P86	Deletion P86
chr11	q14.1 - q22.2	MAML2	mastermind-like 2 (Drosophila)	0.709864			
chr11	q14.1 - q22.2	PICALM	phosphatidylinositol binding clathrin assembly protein	0.709864			
chr11	q14.1 - q14.3	PICALM	phosphatidylinositol binding clathrin assembly protein	0.48336			
chr12	q11 - q13.2	ARID2	AT rich interactive domain 2 (ARID, RFX-like)	0.458262		1.178124	
chr12	q11 - q13.2	ATF1	activating transcription factor 1	0.458262		1.178124	
chr12	q11 - q13.2	HOXC11	homeobox C11	0.458262		1.178124	
chr12	q11 - q13.2	HOXC13	homeobox C13	0.458262		1.178124	
chr12	q11 - q13.2	MLL2	myeloid/lymphoid or mixed-lineage leukemia 2	0.458262		1.178124	
chr12	p12.1 - p11.1	KRAS	v-Ki-ras2 Kirsten rat sarcoma viral oncogene homolog			0.823773	
chr12	q11 - q24.33	ALDH2	aldehyde dehydrogenase 2 family (mitochondrial)			0.670705	
chr12	q11 - q24.33	ARID2	AT rich interactive domain 2 (ARID, RFX-like)			0.670705	
chr12	q11 - q24.33	ATF1	activating transcription factor 1			0.670705	
chr12	q11 - q24.33	BCL7A	B-cell CLL/lymphoma 7A			0.670705	
chr12	q11 - q24.33	BTG1	B-cell translocation gene 1, anti-proliferative			0.670705	
chr12	q11 - q24.33	CDK4	cyclin-dependent kinase 4			0.670705	
chr12	q11 - q24.33	DDIT3	DNA-damage-inducible transcript 3			0.670705	
chr12	q11 - q24.33	HMGA2	high mobility group AT-hook 2			0.670705	
chr12	q11 - q24.33	HNF1A	HNF1 homeobox A			0.670705	
chr12	q11 - q24.33	HOXC11	homeobox C11			0.670705	
chr12	q11 - q24.33	HOXC13	homeobox C13			0.670705	
chr12	q11 - q24.33	LRIG3	leucine-rich repeats and immunoglobulin-like domains 3			0.670705	
chr12	q11 - q24.33	MDM2	Mdm2 p53 binding protein homolog (mouse)			0.670705	
chr12	q11 - q24.33	MLL2	myeloid/lymphoid or mixed-lineage leukemia 2			0.670705	
chr12	q11 - q24.33	NACA	nascent polypeptide-associated complex alpha subunit			0.670705	
chr12	q11 - q24.33	PTPN11	protein tyrosine phosphatase, non-receptor type 11 similar to protein tyrosine phosphatase, non-receptor type 11			0.670705	
chr12	q11 - q24.33	WIF1	WNT inhibitory factor 1			0.670705	
chr12	p13.31	ZNF384	zinc finger protein 384			0.55643	
chr12	q13.3 - q24.33	ALDH2	aldehyde dehydrogenase 2 family (mitochondrial)			0.550591	
chr12	q13.3 - q24.33	BCL7A	B-cell CLL/lymphoma 7A			0.550591	
chr12	q13.3 - q24.33	BTG1	B-cell translocation gene 1, anti-proliferative			0.550591	
chr12	q13.3 - q24.33	CDK4	cyclin-dependent kinase 4			0.550591	
chr12	q13.3 - q24.33	DDIT3	DNA-damage-inducible transcript 3			0.550591	
chr12	q13.3 - q24.33	HMGA2	high mobility group AT-hook 2			0.550591	
chr12	q13.3 - q24.33	HNF1A	HNF1 homeobox A			0.550591	
chr12	q13.3 - q24.33	LRIG3	leucine-rich repeats and immunoglobulin-like domains 3			0.550591	
chr12	q13.3 - q24.33	MDM2	Mdm2 p53 binding protein homolog (mouse)			0.550591	
chr12	q13.3 - q24.33	NACA	nascent polypeptide-associated complex alpha subunit			0.550591	
chr12	q13.3 - q24.33	PTPN11	protein tyrosine phosphatase, non-receptor type 11 similar to protein tyrosine phosphatase, non-receptor type 11			0.550591	
chr12	q13.3 - q24.33	WIF1	WNT inhibitory factor 1			0.550591	
chr13	q21.1 - q34	ERCC5	excision repair cross-complementing rodent repair deficiency, complementation group 5			0.633559	

ADDITIONAL TABLE 2: aCGH data of Low- and High-Passage KCI-MENG1 Cells

Chr	CytoBand	GeneID	Gene name	Amplification P6	Deletion P6	Amplification P86	Deletion P86
chr14	q23.3	GPHN	gephyrin		-0.714782		-0.656267
chr15	q26.1 - q26.3	BLM	Bloom syndrome, RecQ helicase-like	0.490823		1.163907	
chr15	q26.1 - q26.3	CRTC3	CREB regulated transcription coactivator 3	0.490823		1.163907	
chr15	q26.1 - q26.3	IDH2	isocitrate dehydrogenase 2 (NADP+), mitochondrial	0.490823		1.163907	
chr15	q14	C15orf55	chromosome 15 open reading frame 55	0.321231		0.935	
chr15	q25.1 - q26.1	NTRK3	neurotrophic tyrosine kinase, receptor, type 3			0.867012	
chr15	q11.1 - q26.3	BLM	Bloom syndrome, RecQ helicase-like			0.727721	
chr15	q11.1 - q26.3	BUB1B	budding uninhibited by benzimidazoles 1 homolog beta (yeast)			0.727721	
chr15	q11.1 - q26.3	C15orf55	chromosome 15 open reading frame 55			0.727721	
chr15	q11.1 - q26.3	CASC5	cancer susceptibility candidate 5			0.727721	
chr15	q11.1 - q26.3	CRTC3	CREB regulated transcription coactivator 3			0.727721	
chr15	q11.1 - q26.3	FLJ27352	hypothetical LOC145788			0.727721	
chr15	q11.1 - q26.3	HMGN2P46	high mobility group AT-hook 2			0.727721	
chr15	q11.1 - q26.3	IDH2	isocitrate dehydrogenase 2 (NADP+), mitochondrial			0.727721	
chr15	q11.1 - q26.3	NTRK3	neurotrophic tyrosine kinase, receptor, type 3			0.727721	
chr15	q11.1 - q26.3	PML	promyelocytic leukemia similar to promyelocytic leukemia protein isoform 1			0.727721	
chr15	q11.1 - q26.3	TCF12	transcription factor 12			0.727721	
chr15	q11.2 - q24.2	BUB1B	budding uninhibited by benzimidazoles 1 homolog beta (yeast)			0.555837	
chr15	q11.2 - q24.2	C15orf55	chromosome 15 open reading frame 55			0.555837	
chr15	q11.2 - q24.2	CASC5	cancer susceptibility candidate 5			0.555837	
chr15	q11.2 - q24.2	FLJ27352	hypothetical LOC145788			0.555837	
chr15	q11.2 - q24.2	HMGN2P46	high mobility group AT-hook 2			0.555837	
chr15	q11.2 - q24.2	PML	promyelocytic leukemia similar to promyelocytic leukemia protein isoform 1			0.555837	
chr15	q11.2 - q24.2	TCF12	transcription factor 12			0.555837	
chr15	q24.2 - q26.3	BLM	Bloom syndrome, RecQ helicase-like	0.37759			
chr15	q24.2 - q26.3	CRTC3	CREB regulated transcription coactivator 3	0.37759			
chr15	q24.2 - q26.3	IDH2	isocitrate dehydrogenase 2 (NADP+), mitochondrial	0.37759			
chr15	q24.2 - q26.3	NTRK3	neurotrophic tyrosine kinase, receptor, type 3	0.37759			
chr16	p13.11	MYH11	myosin, heavy chain 11, smooth muscle			0.815365	
chr16	p13.3 - p11.1	C16orf75	chromosome 16 open reading frame 75			0.503333	
chr16	p13.3 - p11.1	CIITA	class II, major histocompatibility complex, transactivator			0.503333	
chr16	p13.3 - p11.1	CREBBP	CREB binding protein			0.503333	
chr16	p13.3 - p11.1	ERCC4	excision repair cross-complementing rodent repair deficiency, complementation group 4			0.503333	
chr16	p13.3 - p11.1	FUS	fusion (involved in t(1216) in malignant liposarcoma)			0.503333	
chr16	p13.3 - p11.1	IL21R	interleukin 21 receptor			0.503333	
chr16	p13.3 - p11.1	MYH11	myosin, heavy chain 11, smooth muscle			0.503333	
chr16	p13.3 - p11.1	PALB2	partner and localizer of BRCA2			0.503333	
chr16	p13.3 - p11.1	RUNDC2A	ribophorin I			0.503333	
chr16	p13.3 - p11.1	SNX29	sorting nexin 29			0.503333	
chr16	p13.3 - p11.1	SOCS1	suppressor of cytokine signaling 1			0.503333	

ADDITIONAL TABLE 2: aCGH data of Low- and High-Passage KCI-MENG1 Cells

Chr	CytoBand	GeneID	Gene name	Amplification P6	Deletion P6	Amplification P86	Deletion P86
chr16	p13.3 - p11.1	TNFRSF17	tumor necrosis factor receptor superfamily, member 17			0.503333	
chr16	p13.3 - p11.1	TSC2	tuberous sclerosis 2			0.503333	
chr16	q11.2 - q24.3	CBFA2T3	cancer susceptibility candidate 5			0.49455	
chr16	q11.2 - q24.3	CBFB	core-binding factor, beta subunit			0.49455	
chr16	q11.2 - q24.3	CDH1	cadherin 1, type 1, E-cadherin (epithelial)			0.49455	
chr16	q11.2 - q24.3	CDH11	cadherin 11, type 2, OB-cadherin (osteoblast)			0.49455	
chr16	q11.2 - q24.3	CYLD	cylindromatosis (turban tumor syndrome)			0.49455	
chr16	q11.2 - q24.3	FANCA	Fanconi anemia, complementation group A			0.49455	
chr16	q11.2 - q24.3	HERPUD1	H3 histone, family 3B (H3.3B) H3 histone, family 3A pseudogene			0.49455	
chr16	q11.2 - q24.3	MAF	v-maf musculoaponeurotic fibrosarcoma oncogene homolog (avian)			0.49455	
chr17	p13.3 - p11.1	FLCN	folliculin			0.534252	
chr17	p13.3 - p11.1	GAS7	growth arrest-specific 7			0.534252	
chr17	p13.3 - p11.1	MAP2K4	mitogen-activated protein kinase kinase 4			0.534252	
chr17	p13.3 - p11.1	PER1	period homolog 1 (Drosophila)			0.534252	
chr17	p13.3 - p11.1	RABEP1	rabaptin, RAB GTPase binding effector protein 1			0.534252	
chr17	p13.3 - p11.1	SPECC1	suppressor of cytokine signaling 1			0.534252	
chr17	p13.3 - p11.1	TP53	tumor protein p53			0.534252	
chr17	p13.3 - p11.1	USP6	ubiquitin specific peptidase 6 (Tre-2 oncogene)			0.534252	
chr17	p13.3 - p11.1	YWHAE	similar to 14-3-3 protein epsilon (14-3-3E) (Mitochondrial import stimulation factor L subunit) (MSF L) tyrosine 3-monooxygenase/tryptophan 5-monooxygenase activation protein, epsilon polypeptide			0.534252	
chr17	q11.1 - q25.3	ASPSCR1	aryl hydrocarbon receptor nuclear translocator			0.514077	
chr17	q11.1 - q25.3	BRCA1	breast cancer 1, early onset			0.514077	
chr17	q11.1 - q25.3	BRIP1	BRCA1 interacting protein C-terminal helicase 1			0.514077	
chr17	q11.1 - q25.3	CANT1	calcium activated nucleotidase 1			0.514077	
chr17	q11.1 - q25.3	CD79B	CD79b molecule, immunoglobulin-associated beta			0.514077	
chr17	q11.1 - q25.3	CDK12	Cdc2-related kinase, arginine/serine-rich			0.514077	
chr17	q11.1 - q25.3	CLTC	clathrin, heavy chain (Hc)			0.514077	
chr17	q11.1 - q25.3	COL1A1	collagen, type I, alpha 1			0.514077	
chr17	q11.1 - q25.3	DDX5	DEAD (Asp-Glu-Ala-Asp) box polypeptide 5			0.514077	
chr17	q11.1 - q25.3	ERBB2	v-erb-b2 erythroblastic leukemia viral oncogene homolog 2, neuro/glioblastoma derived oncogene homolog (avian)			0.514077	
chr17	q11.1 - q25.3	ETV4	ets variant 4			0.514077	
chr17	q11.1 - q25.3	HLF	hepatic leukemia factor			0.514077	
chr17	q11.1 - q25.3	LASP1	LIM and SH3 protein 1			0.514077	
chr17	q11.1 - q25.3	MLLT6	myeloid/lymphoid or mixed-lineage leukemia (trithorax homolog, Drosophila) translocated to, 6			0.514077	
chr17	q11.1 - q25.3	MSI2	musashi homolog 2 (Drosophila)			0.514077	
chr17	q11.1 - q25.3	NF1	neurofibromin 1			0.514077	
chr17	q11.1 - q25.3	PRKAR1A	perforin 1 (pore forming protein)			0.514077	
chr17	q11.1 - q25.3	RARA	retinoic acid receptor, alpha			0.514077	

ADDITIONAL TABLE 2: aCGH data of Low- and High-Passage KCI-MENG1 Cells

Chr	CytoBand	GeneID	Gene name	Amplification P6	Deletion P6	Amplification P86	Deletion P86
chr17	q11.1 - q25.3	RNF213	ring finger protein 213			0.514077	
chr17	q11.1 - q25.3	SEPT9	similar to succinate dehydrogenase complex, subunit D, integral membrane protein succinate dehydrogenase complex, subunit D, integral membrane protein			0.514077	
chr17	q11.1 - q25.3	SRSF2	SLIT-ROBO Rho GTPase activating protein 3			0.514077	
chr17	q11.1 - q25.3	SUZ12	suppressor of zeste 12 homolog (Drosophila)			0.514077	
chr17	q11.1 - q25.3	TAF15	TAF15 RNA polymerase II, TATA box binding protein (TBP)-associated factor, 68kDa			0.514077	
chr19	q13.41 - q13.43	TFPT	TCF3 (E2A) fusion partner (in childhood Leukemia)			0.574435	
chr19	q13.41 - q13.43	ZNF331	zinc finger protein 331			0.574435	
chr19	q11 - q13.43	AKT2	v-akt murine thymoma viral oncogene homolog 2			0.425134	
chr19	q11 - q13.43	BCL3	B-cell CLL/lymphoma 3			0.425134	
chr19	q11 - q13.43	CBLC	Cas-Br-M (murine) ecotropic retroviral transforming sequence c			0.425134	
chr19	q11 - q13.43	CCNE1	cyclin E1			0.425134	
chr19	q11 - q13.43	CD79A	CD79a molecule, immunoglobulin-associated alpha			0.425134	
chr19	q11 - q13.43	CEBPA	CCAAT/enhancer binding protein (C/EBP), alpha			0.425134	
chr19	q11 - q13.43	CIC	capicua homolog (Drosophila)			0.425134	
chr19	q11 - q13.43	ERCC2	excision repair cross-complementing rodent repair deficiency, complementation group 2			0.425134	
chr19	q11 - q13.43	KLK2	kallikrein-related peptidase 2			0.425134	
chr19	q11 - q13.43	PPP2R1A	peroxisome proliferator-activated receptor gamma			0.425134	
chr19	q11 - q13.43	TFPT	TCF3 (E2A) fusion partner (in childhood Leukemia)			0.425134	
chr19	q11 - q13.43	ZNF331	zinc finger protein 331			0.425134	
chr19	p13.3 - p11	BRD4	bromodomain containing 4			0.411723	
chr19	p13.3 - p11	CRTC1	CREB regulated transcription coactivator 1			0.411723	
chr19	p13.3 - p11	DNM2	dynamin 2			0.411723	
chr19	p13.3 - p11	ELL	elongation factor RNA polymerase II			0.411723	
chr19	p13.3 - p11	JAK3	Janus kinase 3			0.411723	
chr19	p13.3 - p11	LYL1	lymphoblastic leukemia derived sequence 1			0.411723	
chr19	p13.3 - p11	SMARCA4	SWI/SNF Related, Matrix Associated, Actin Dependent Regulator of Chromatin, Subfamily A, Member 4			0.411723	
chr19	p13.3 - p11	TPM4	tropomyosin 4			0.411723	
chr19	p13.3	STK11	serine/threonine kinase 11		-1.376387		-3.097343
chr20	q13.2 - q13.33	GNAS	GNAS complex locus	0.470244		1.148625	
chr20	q13.2 - q13.33	SS18L1	synovial sarcoma translocation gene on chromosome 18-like 1	0.470244		1.148625	
chr20	q11.21 - q13.33	ASXL1	additional sex combs like 1 (Drosophila)	0.331638		0.958835	
chr20	q11.21 - q13.33	GNAS	GNAS complex locus	0.331638		0.958835	
chr20	q11.21 - q13.33	MAFB	v-maf musculoaponeurotic fibrosarcoma oncogene homolog B (avian)	0.331638		0.958835	
chr20	q11.21 - q13.33	SDC4	syndecan 4	0.331638		0.958835	
chr20	q11.21 - q13.33	SS18L1	synovial sarcoma translocation gene on chromosome 18-like 1	0.331638		0.958835	
chr20	q11.21 - q13.33	TOP1	topoisomerase (DNA) I	0.331638		0.958835	
chr20	q11.21 - q13.2	ASXL1	additional sex combs like 1 (Drosophila)			0.859745	

ADDITIONAL TABLE 2: aCGH data of Low- and High-Passage KCI-MENG1 Cells

Chr	CytoBand	GeneID	Gene name	Amplification P6	Deletion P6	Amplification P86	Deletion P86
chr20	q11.21 - q13.2	MAFB	v-maf musculoaponeurotic fibrosarcoma oncogene homolog B (avian)			0.859745	
chr20	q11.21 - q13.2	SDC4	syndecan 4			0.859745	
chr20	q11.21 - q13.2	TOP1	topoisomerase (DNA) I			0.859745	
chrX	p11.22 - p11.21	KDM5C	lysine (K)-specific demethylase 5C			0.90204	
chrX	p11.22 - p11.21	SSX2	synovial sarcoma, X breakpoint 2 synovial sarcoma, X breakpoint 5, synovial sarcoma, X breakpoint 2B			0.90204	
chrX	p22.33 - p11.1	BCOR	BCL6 co-repressor			0.58112	
chrX	p22.33 - p11.1	CRLF2	cytokine receptor-like factor 2			0.58112	
chrX	p22.33 - p11.1	GATA1	GATA binding protein 1 (globin transcription factor 1)			0.58112	
chrX	p22.33 - p11.1	KDM5C	lysine (K)-specific demethylase 5C			0.58112	
chrX	p22.33 - p11.1	KDM6A	lysine (K)-specific demethylase 6A			0.58112	
chrX	p22.33 - p11.1	P2RY8	purinergic receptor P2Y, G-protein coupled, 8			0.58112	
chrX	p22.33 - p11.1	SSX1	synovial sarcoma, X breakpoint 8 synovial sarcoma, X breakpoint 1			0.58112	
chrX	p22.33 - p11.1	SSX2	synovial sarcoma, X breakpoint 2 synovial sarcoma, X breakpoint 5, synovial sarcoma, X breakpoint 2B			0.58112	
chrX	p22.33 - p11.1	SSX4	synovial sarcoma, X breakpoint 4 synovial sarcoma, X breakpoint 4B			0.58112	
chrX	p22.33 - p11.1	TFE3	transcription factor binding to IGHM enhancer 3			0.58112	
chrX	p22.33 - p11.1	WAS	Wiskott-Aldrich syndrome (eczema-thrombocytopenia)			0.58112	
chrX	p22.33 - p11.1	ZRSR2	zinc finger (CCCH type), RNA-binding motif and serine/arginine rich 2			0.58112	
chrX	q11.1 - q12	FAM123B	enhancer of zeste homolog 2 (Drosophila)			0.366791	
chrX	q11.1 - q12	MSN	moesin			0.366791	
chrX	p11.22 - p11.1	KDM5C	lysine (K)-specific demethylase 5C	0.301484			
chrX	p11.22 - p11.1	SSX2	synovial sarcoma, X breakpoint 2 synovial sarcoma, X breakpoint 5, synovial sarcoma, X breakpoint 2B	0.301484			
